# Scalable and Robust Artificial Intelligence for Spine Alignment Assessment: Multicenter Study Enabled by Real-Time Data Transformation

**DOI:** 10.2196/78396

**Published:** 2026-03-20

**Authors:** Guilin Chen, Nan Meng, Yipeng Zhuang, Zhe Chen, Zhen Bian, Zhaoyang Gong, Jiawei Shi, Tao Huang, Xihe Kuang, Pengyu Lu, Cong Nie, Qifeng Yu, Zefu Chen, Hui Jiang, Zhongmin Zhang, Chaojun Zheng, Yu Liang, Nan Wu, Jason Pui Yin Cheung, Jianguo Zhang, Teng Zhang

**Affiliations:** 1 Department of Orthopaedic Surgery, State Key Laboratory of Complex Severe and Rare Diseases, Beijing Key of Big Data Innovation and Application for Skeletal Health Medical Care, Key Laboratory of Big Data for Spinal Deformities Peking Union Medical College Hospital, Peking Union Medical College and Chinese Academy of Medical Sciences Beijing, 100730 China; 2 Department of Orthopaedics and Traumatology Li Ka Shing Faculty of Medicine The University of Hong Kong HONG KONG SAR China (Hong Kong); 3 Department of Orthopaedics Shanghai Jiaotong University School of Medicine Ruijin Hospital Shanghai, 200025 China; 4 Department of Paediatric Orthopaedics Beijing Jishuitan Hospital Beijing, 100035, Beijing China; 5 Department of Orthopaedics Huashan Hospital, Fudan University Shanghai, 200040 China; 6 Department of Orthopaedics Division of Spine Surgery Nanfang Hospital, Southern Medical University Guangzhou, Guangdong, 510515 China

**Keywords:** adolescent idiopathic scoliosis, multicentre validation, data heterogeneity, artificial intelligence, data transformation

## Abstract

**Background:**

Artificial intelligence (AI) has shown promise for automating spinal alignment assessment in adolescent idiopathic scoliosis (AIS). However, AI models typically exhibit reduced accuracy and robustness when deployed across multiple medical centers due to variability in imaging protocols and data characteristics, potentially compromising clinical diagnosis and treatment decisions.

**Objective:**

This study aimed to develop a real-time, plug-and-play data transformation method to enhance the robustness of deep learning models against data heterogeneity in radiographs, thereby improving their performance in assessing AIS across multiple medical centers.

**Methods:**

In this retrospective multicenter study, 3899 full-spine radiographs from 7 hospitals (2 from Hong Kong and 5 from Mainland China), collected between January 2012 and August 2024, were included. Data from 2 hospitals in Hong Kong (n=3034) were used for model training and internal validation, while radiographs from the 5 mainland hospitals (n=865) formed 5 independent external validation datasets. A novel pixel intensity–based data transformation method was developed to standardize image contrast and brightness across datasets and integrated into the model training process to enhance our previously developed AI model, SpineHRNet+. The enhanced model's accuracy and robustness for cobb angle (CA) prediction and severity classification were evaluated using both internal and external datasets. Data heterogeneity across centers was quantified by brightness and contrast differences. CA prediction accuracy was evaluated using residual analysis, linear regression (coefficient of determination [*R*²]), and Bland-Altman analyses. Model performance for disease severity classification was assessed using sensitivity, specificity, precision, negative predictive value, accuracy, and confusion matrix analysis. The transformation method aligns pixel intensity distributions across datasets using statistical profiling and optimization, ensuring consistent image characteristics while preserving anatomical integrity.

**Results:**

The developed data transformation method significantly reduced contrast variability between datasets, improving consistency in image characteristics and enabling more reliable AI analysis. The enhanced SpineHRNet+ achieved consistent and accurate CA predictions across external validation datasets, with mean prediction errors within 4° (SD 3.12°), and maintained an *R*² greater than 0.90 for all centers. The sensitivity and negative predictive value for disease severity grading improved to 90.18% and 93.16%, respectively. Bland-Altman analyses demonstrated robust agreement, with 95% limits of agreement within 7.51° across all datasets.

**Conclusions:**

The proposed data transformation approach effectively addressed data heterogeneity, significantly improving the accuracy and robustness of SpineHRNet+ in multicenter AIS assessments. The real-time processing capability and preservation of anatomical integrity underscore the method’s clinical practicality, enabling scalable and reliable AI applications in diverse health care environments.

## Introduction

Adolescent idiopathic scoliosis (AIS) is one of the most common spinal deformities in adolescents, affecting approximately 2.2% of boys and 4.8% of girls globally [1,2]. Without timely diagnosis and intervention, AIS may progress rapidly to severe spinal curvatures [3], potentially leading to cosmetic concerns, back pain, and reduced cardiopulmonary function [4]. The Cobb angle (CA), a measurement derived from spinal radiographs, is considered the gold standard for quantitatively assessing scoliosis severity and guiding clinical decision-making such as bracing or surgical intervention [5]. However, accurate measurement of the CA is inherently laborious and requires considerable clinical expertise, often exhibiting inter- and intra-observer variability ranging between 4.9° to 10° [6,7]. This variability is particularly pronounced in multicenter settings, given differences in patient positioning, clinical protocols, and assessment practices across institutions [8,9]. Despite promising results in single-center studies, existing AI models lack mechanisms to adapt to intercenter variability, highlighting a critical gap in robust multicenter deployment. Unlike prior domain adaptation or generative approaches that often require paired data or introduce distortions, our method offers a novel unsupervised pixel-level transformation that preserves anatomical integrity while standardizing image characteristics.

Advances in digital technologies, particularly artificial intelligence (AI), have shown promising potential to improve the consistency and efficiency of AIS assessment [10-13]. Recent AI-based models have demonstrated high reliability and accuracy in CA prediction and severity grading when validated on single-center datasets [14,15]. Despite these successes, widespread clinical adoption remains challenging due to heterogeneity in clinical data collected across different health care facilities [16]. Variability in spinal radiograph quality, brightness, contrast, and pixel intensity distributions, stemming from differences in the medical equipment, acquisition protocols, and post-processing methods, substantially hampers the generalizability of AI models when applied across external institutions [17-19]. These discrepancies limit the real-world applicability and use of AI models, especially in resource-constrained clinical environments, where the absence of uniform standards and variations in imaging practices further exacerbate the performance degradation of AI diagnostic tools. Additionally, spine surgeons commonly capture radiographs informally, either using smartphone cameras or taking screenshots directly from computer monitors during routine clinical practice. Such practices markedly degrade image quality and exacerbate inaccuracies in current AI-based automated analysis methods, undermining the reliability of spinal alignment evaluations. These practical challenges highlight an urgent need for methods capable of adapting to variations in image quality and acquisition techniques, ensuring robust and dependable clinical assessments across diverse health care settings.

To address the limitations of existing AI models in robustness and generalizability, this study introduces a plug-and-play data transformation approach harmonizing characteristics at the pixel-level to effectively mitigate differences among multicenter datasets. The system operates without calibration using a default reference profile, but performance improves with minimal calibration. This computationally efficient approach can be easily integrated into the training or fine-tuning processes of existing AI models, significantly enhancing their robustness and stability in handling data from diverse sources. Using this method, we enhanced SpineHRNet+, an AI model originally trained on a single-center dataset, and evaluated its accuracy and robustness in predicting the CA through internal validation and 5 independent external datasets. By improving model performance across heterogeneous data with minimal computational overhead and data requirements, this research paves the way for reliable clinical implementation of AI-assisted AIS diagnosis and assessment across diverse clinical settings. Previous efforts to address data heterogeneity have included statistical harmonization techniques, such as histogram equalization, intensity normalization, and feature distribution alignment [16,17]. Domain adaptation methods like covariance matching and structure-preserving projection have also been explored to reduce intercenter variability. While these approaches offer theoretical robustness, many require extensive preprocessing, paired data, or high computational resources, limiting their scalability in clinical environments. In contrast, our method uses unsupervised pixel intensity alignment using histogram-based statistical profiling, offering a lightweight, anatomically preserving, and real-time solution suitable for multicenter deployment.

## Methods

### Study Design and Participants

Deidentified posteroanterior whole-spine radiographs of AIS patients (inclusion and exclusion criteria detailed in Section 2 in Multimedia Appendix 1) were gathered from 5 tertiary hospitals (n=865) in Mainland China and 2 hospitals in Hong Kong China (n=3034), spanning from January 1, 2012, to August 8, 2024. Demographic data, including age, sex, height, weight, and BMI, as well as the radiograph acquisition protocols, were extracted from medical records at each center. Radiographs from Queen Mary Hospital (QMH) and DKCH (**Duchess of Kent Children’s Hospital at Sandy Bay**; QMH&DKCH cohort) were specifically used for model training and internal validation: 2770 patients were randomly selected for the training set, and 264 patients were selected using a stratified hold-out approach to ensure consistent distribution of severity and curve types. Data from the remaining 5 hospitals formed independent external validation datasets: Peking Union Medical College Hospital (PUMCH) cohort, First Affiliated Hospital, Zhejiang University School of Medicine (FAH) cohort, Qilu Hospital of Shandong University (QLH) cohort, Shandong Provincial Hospital (SDPH) cohort, and Shanghai Huashan Hospital (HSH) cohort. Radiographs from QMH and DKCH were used for model training and internal validation.

### Data Collection and Preparation

Radiographs were acquired using site-specific imaging setups and workflows. Specifically, QMH&DKCH and SDPH used EOS systems (EOSedge at QMH&DKCH; EOS Imaging System at RJH) for biplanar whole-spine radiographs. PUMCH used a Philips DigitalDiagnost system, sequentially capturing images of different spinal regions and stitching them together to form whole-spine radiographs. FAH and HSH used GE health care system (Discovery XR656 at FAH and Optima XR646 HD at HSH) to acquire posteroanterior whole-spine radiographs, while QLH used multiple imaging devices, such as the KODAK DIRECTVIEW DR 7500, Canon DR, CARESTREAM DRX-EVOLUTION, and EOS systems. Notable variability in critical imaging parameters, such as kilovolt peak, exposure time, and area dose product, further highlighted dataset heterogeneity (Table S1 in Multimedia Appendix 1). Detailed imaging protocols are provided in Section 1 in Multimedia Appendix 1.

All radiographs were annotated according to standardized procedures using the AlignProCARE system, marking 72 vertebral endplate landmarks from the seventh cervical vertebra (C7) to the fifth lumbar vertebra (L5). At QMH&DKCH, landmarks were initially annotated by 2 junior clinicians with at least 2 years of clinical experience, and subsequently verified by a senior spine specialist with more than 20 years of experience. To ensure annotation consistency across centers, external annotations were cross-checked by senior spine specialists, and a subset of 10% randomly selected radiographs from each center were reannotated and reviewed by an independent expert panel to verify intercenter consistency. The annotated landmarks served as ground truth (GT) for model training and evaluation.

The coronal CA was used as the primary metric for evaluating spinal alignment. Deformities with CA exceeding 40° were deemed severe, those ranging from 20° to 40° were labeled as moderate, and angles from 0° to 20° were identified as normal-mild. The criteria for assessing the severity of spinal deformities and their corresponding clinical interventions are detailed in Table S2 in Multimedia Appendix 1. The classification of curve types was determined by the location of the curve apex: thoracic (T) curves were identified when the apex was situated between the T1 and T11 vertebrae, while T or TL curves were recognized if the apex fell between the T12 and the L5 vertebrae.

### Model Development and Multicenter Validation

**Figure 1:**
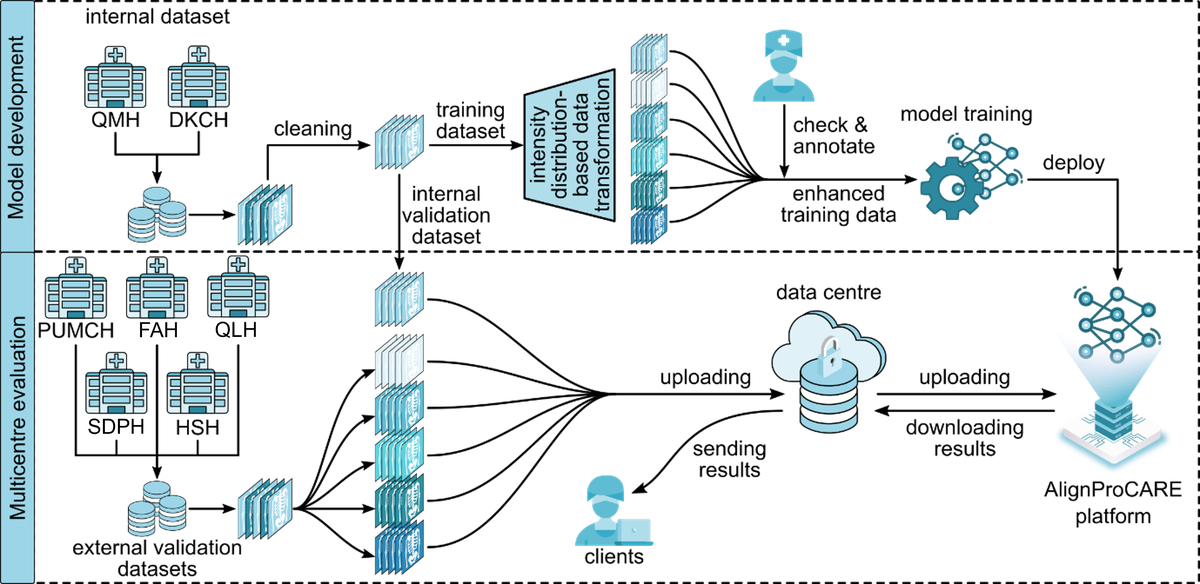
The workflow diagram of model development and real-world application. In model development stage, radiographs collected from 2 local clinics (QMH and DKCH) were first augmented by the proposed data transformation method and then used for model training. Subsequently, the well-trained model was deployed on our online platform mskalign AlignProCARE. In the real-world application scenario, X-rays obtained from 5 external centres were used as external testing data and the ones collected from QMH and DKCH were used as internal validation data to comprehensively evaluate the performance of our model. DKCH: Duchess of Kent Children's Hospital; FAH: First Affiliated Hospital; HSH: Shanghai Huashan Hospital; PUMCH: Peking Union Medical College Hospital; QLH: Qilu Hospital of Shandong University; QMH: Queen Mary hospital; SDPH: Shandong Provincial Hospital.

We used SpineHRNet+ [15], a previously validated deep learning model, and enhanced it through the proposed data transformation method. The overall workflow consisted of 2 phases: model development (with data transformation integration) and multicenter validation (Figure 1). During model development, we applied the data transformation method to the QMH&DKCH training set, creating simulated radiographs that mimic pixel-level characteristics from external centers. SpineHRNet+ was trained and fine-tuned using both original and transformed datasets to improve generalizability. Subsequently, the enhanced model was deployed via the AlignProCARE system for clinical evaluation across multiple institutions. During external validation, each medical center uploaded radiographs through locally installed AlignProCARE software or applications; images were processed remotely by the enhanced model, and results were returned to the respective centers for clinical analysis.

To preserve transformation diversity, reference images from each external center were randomly sampled during augmentation. Each training image was transformed multiple times using different reference distributions, generating a diverse set of simulated radiographs. Augmented samples were randomly mixed with original images during training, without weighting, to ensure balanced exposure and prevent overfitting to any specific external style.

### Intensity Distribution–Based Data Transformation

To address data heterogeneity in clinical data collected from different institutions, we developed a data transformation approach to standardize differences observed across patient spinal radiographs from multiple centers. Our method works by analyzing and aligning the quantitative characteristics, such as brightness and contrast, present in clinical images from different hospitals. Specifically, we first calculated detailed statistical profiles representing the distribution of intensity values for each institution. Then, using an optimization technique, we systematically adjusted each patient's data to align closely with a reference standard.

**Figure 2:**
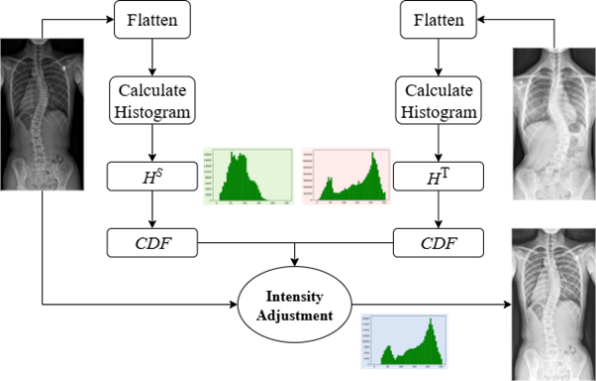
Visual results of intensity distribution-based data transform algorithm. CDF: cumulative distribution function.

The transformation function is based on histogram matching, where the cumulative distribution function (CDF) of the source image is aligned to that of a target image. The optimization objective minimizes the absolute difference between the CDFs:


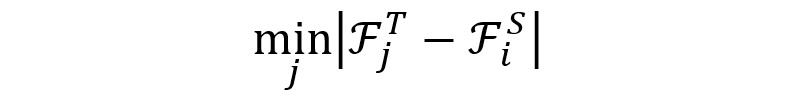


for each intensity level *i*, where *F^S^* and *F^T^* are the CDFs of the source and target images, respectively. The mapping is monotonic and preserves anatomical integrity by avoiding spatial distortion. Detailed pseudocode is provided in Algorithm S1 in Multimedia Appendix 1. Figure 2 illustrate the transformation workflow. This alignment process was carefully designed to preserve essential clinical and anatomical details, ensuring that no critical diagnostic information was altered or lost (Algorithm S1 and Figure S1 in Multimedia Appendix 1). Brightness differences are reported in normalized pixel intensity units ranging from 0 to 255. Histogram spread refers to the SD of pixel intensity values across the image, indicating contrast variability.

Conceptually, the algorithm computes the histogram of pixel intensities for each image and aligns it to a reference distribution using a monotonic mapping function. This mapping is derived via histogram matching and optimized to minimize brightness and contrast discrepancies while preserving anatomical structures. The transformation is applied in real-time and does not require paired training data. Unlike conventional histogram matching applied as a preprocessing step, our method dynamically samples reference distributions from multiple centers and integrates transformation into model training, enabling real-time adaptation and improved robustness. The procedure is computationally efficient, allowing rapid integration into routine clinical workflows, even when handling large numbers of patient records (computational complexity *O*(*n* log n); Multimedia Appendix 1 Section 3.3). For simplicity, the AI model incorporating this transformation technique is termed the “enhanced model” in subsequent sections.

### Statistical Analysis

To evaluate the accuracy of CA predictions, we calculated the mean (SD) of the absolute differences between predicted and GT values. The distribution of these prediction errors was assessed using descriptive statistics, including the first quartile (Q1), median, third quartile (Q3), SD, and IQR. Linear regression analysis was used to assess the correlation between predicted and GT values, quantified by the coefficient of determination (*R*²). Bland-Altman analysis was used to measure the agreement between model predictions and GT, reporting the 95% limits of agreement (LoA). Paired *t* tests were used to compare the absolute prediction errors of the 2 models on the same set of images, controlling for patient-specific anatomical variability.

Performance in disease severity grading and spinal curve identification was evaluated using confusion matrices. Sensitivity, specificity, precision, negative predictive value (NPV), and accuracy were calculated, each with a corresponding 95% CI.

All statistical analyses were conducted using Python (v3.8) and several related packages, including NumPy [20] (v1.18.5), SciPy [21] (v1.5.2), Ptitprince [22] (v0.2.6), pandas [23] (v1.1.3), seaborn [24] (v0.11.0), and Matplotlib [25] (v3.3.2).

### Ethical Considerations

This multicenter retrospective cohort study adhered to the Declaration of Helsinki and the Standards for Reporting of Diagnostic Accuracy Studies reporting guidelines and was registered at website of Clinical Trials (Registration No. NCT06711757). Ethical approval was obtained from the Institutional Review Boards (IRBs) of all participating medical centers, including QMH and DKCH (IRB No. UW22-270); PUMCH (IRB No. IRB-B473); FAH (IRB No. IIT20230248B-R1); QLH (IRB No. QLCR20240818); SDPH (IRB No. AF-SW-370-001); and HSH (IRB No. KY2022-570). As this is a retrospective study using previously collected and anonymized patient data, the IRB has waived the requirement for informed consent. No compensation was provided to participants, as no prospective recruitment or study visits were conducted. All patient data used in this study were deidentified prior to analysis. No identifiable personal information was accessed or stored, ensuring full compliance with privacy and confidentiality standards.

## Results

### Overview

All eligible patients with AIS from QMH and DKCH recruited between December 2019 and August 2024 were included, forming a training cohort of 2770 participants (mean 14, SD 1.9 years; 75.3% female; Table 1). An additional 264 consecutive AIS patients recruited from September 2023 to March 2024 served as an internal validation cohort (mean 16.7, SD 1.2 years; 78% female; Table 1). Overall, the internal dataset (n=3034) included 910 patients with normal-mild deformities, 1,624 with moderate deformities, and 236 with severe deformities (Table 1).

**Table 1:** Demographic information of cohorts from different centers.

Demographic information			Training and internal validation datasets (n=3034)				External validation datasets (n=865)								
			Training dataset		Internal validation dataset		External validation dataset 1 (PUMCH^a)^		External validation dataset 2 (FAH^b^)		External validation dataset 3 (QLH^c^)		External validation dataset 4 (SDPH^d^)		External validation dataset 5 (HSH^e^)
							
**Patients**			2770		264		314		105		134		136		176
	Mean age (SD), years	14 (1.9)		16.7 (1.2)		14.8 (2.8)		15.9 (2.7)		14 (2.6)		14.3 (2.8)		15.4 (3.4)	
	Mean max CA^f^ (SD), degree	25 (10.8)		24.5 (9.1)		46.6 (13)		18.7 (10.2)		28.2 (14.3)		20.4 (12.2)		25.1 (16)	
	Mean BMI (SD), kg/m^2^	18.9 (14.8)		18.7 (3.3)		18.4 (3.3)		N/A^g^		N/A		N/A		22.6 (3.7)	
**Sex, n (%)**															
	Female	2085 (75.3)		206 (78)		277 (88.2)		74 (70.5)		81 (60.4)		97 (71.3)		109 (61.9)	
	Male	685 (24.7)		58 (22)		37 (11.8)		31 (29.5)		53 (39.6)		39 (28.7)		67 (38.1)	
**Curve type, n**															
	Thoracic	2497		244		287		76		45		48		86	
	Thoracolumbar or lumbar	2045		193		283		41		111		116		129	
**Severity, n**															
	Normal-mild	910		100		4		56		41		75		79	
	Moderate	1624		147		90		46		72		52		61	
	Severe	236		17		220		3		21		9		36	

^a^PUMCH: Peking Union Medical College Hospital.

^b^FAH: First Affiliated Hospital, Zhejiang University School of Medicine.

^c^QLH: Qilu Hospital of Shandong University.

^d^SDPH: Shandong Provincial Hospital.

^e^HSH: Shanghai Huashan Hospital.

^f^CA: cobb angle.

^g^N/A: not available.

In total, 5 independent external cohorts comprising a total of 865 patients were recruited from the following hospitals: PUMCH (n=314; mean 14.8, SD 2.8 years; 88.2% female), FAH (n=105; mean 15.9, SD 2.7 years; 70.5% female), QLH (n=134; mean 14, SD 2.6 years; 60.4% female), SDPH (n=136; mean 14.3, 2.8 years; 71.3% female), and HSH (n=176; mean 15.4, SD 3.4 years; 61.9% female). Disease severity across these external datasets comprised 255 normal-mild cases, 321 moderate cases, and 289 severe cases (Table 1). Center-specific equipment and protocols were used when collecting each external dataset (Table S1 in Multimedia Appendix 1). The initial differences in histogram spread and brightness values between the internal and external datasets were 9.6×10-4 and 8.71, respectively; after applying the data transformation algorithm, these differences were reduced to 0.4×10-4 and 0.47 (Table S3 in Multimedia Appendix 1).

### CA Prediction

Table 2 summarizes the CA prediction performance of both the original and enhanced models. In the internal dataset (QMH&DKCH cohort), the enhanced model improved prediction accuracy, reducing the mean absolute error by 0.13° (SD 0.27°) for thoracic curves (enhanced: 2.90°, SD 1.95° vs original: 3.03°, SD 2.22°]), and by 0.21°, SD 0.28°) for thoracolumbar or lumbar curves (enhanced: 2.54°, SD 1.86° vs original: 2.75°, SD 2.14°). Across all independent external datasets, the enhanced model reduced the mean absolute prediction error by 1.35° (SD 1.72°) in thoracic curves (enhanced: 4.03°, SD 3.12° vs original: 5.38°, SD 4.83°), and by 1.45° (SD 1.74°) in thoracolumbar or lumbar curves (enhanced: 3.75°, SD 2.94° vs original: 5.21°, SD 4.68°). To assess the significance of performance improvements, paired *t* tests were conducted comparing the prediction errors of the original and enhanced models across all datasets. The results confirmed statistically significant improvements (*P*<.05) across all external cohorts, except for PUMCH's thoracic group, supporting the robustness of our approach.

**Table 2:** Quantitative comparison of absolute error in cobb angle prediction between original and enhanced models on internal and external validation datasets.

Cohorts				QMH^a^ and DKCH^b^		PUMCH^c^		FAH^d^		QLH^e^		SDPH^f^		HSH^g^		Overall (external)
**Thoracic**																
	**Cobb angle difference (degree)**															
		Original model, mean (SD)	3.03 (2.22)		4.08 (3.25)		5.35 (3.73)		5.46 (4.57)		6.88 (7.88)		5.15 (4.73)		5.38 (4.83)	
		Enhanced model, mean (SD)	2.90 (1.95)		3.73 (2.36)		3.77 (2.71)		3.84 (2.76)		4.53 (4.66)		4.30 (3.09)		4.03 (3.12)	
		Improvement (degree), mean (SD)	0.13 (0.27)		0.35 (0.89)		1.58 (1.02)		1.62 (1.81)		2.35 (3.22)		0.85 (1.64)		1.35 (1.72)	
		*P* value	.46		.21		<.01		<.01		<.01		.03		—^h^	
**Thoracolumbar or lumbar**																
	**Cobb angle difference (degree)**															
		Original model, mean (SD)	2.75 (2.14)		4.41 (3.81)		5.63 (5.42)		5.72 (4.56)		5.52 (4.78)		4.75 (4.82)		5.21 (4.68)	
		Enhanced model, mean (SD)	2.54 (1.86)		2.76 (2.08)		4.30 (3.31)		3.81 (2.97)		4.10 (2.93)		3.80 (3.40)		3.75 (2.94)	
		Improvement (degree), mean (SD)	0.21 (0.28)		1.65 (1.73)		1.33 (2.11)		1.91 (1.59)		1.42 (1.85)		0.95 (1.42)		1.45 (1.74)	
		*P* value	.19		<.01		<.01		<.01		<.01		.04		—	

^a^QMH: Queen Mary Hospital.

^b^DKCH: Duchess of Kent Children’s Hospital at Sandy Bay.

^c^PUMCH: Peking Union Medical College Hospital.

^d^FAH: First Affiliated Hospital, Zhejiang University School of Medicine.

^e^QLH: Qilu Hospital of Shandong University.

^f^SDPH: Shandong Provincial Hospital.

^g^HSH: Shanghai Huashan Hospital.

^h^Not available.

CA prediction error distributions (Table S4 in Multimedia Appendix 1) demonstrated that in the internal validation dataset, the SD of prediction errors for T curves was reduced by 0.97, and the IQR decreased by 1.93 when using the enhanced model. For thoracolumbar or lumbar curves, the SD was reduced by 1.17, and the IQR decreased by 1.77. Across all independent external datasets, the average SD for T curves decreased by 1.31, and the IQR by 1.17, while for thoracolumbar or lumbar curves, the average SD was reduced by 1.47, and the IQR by 0.80.

**Figure 3:**
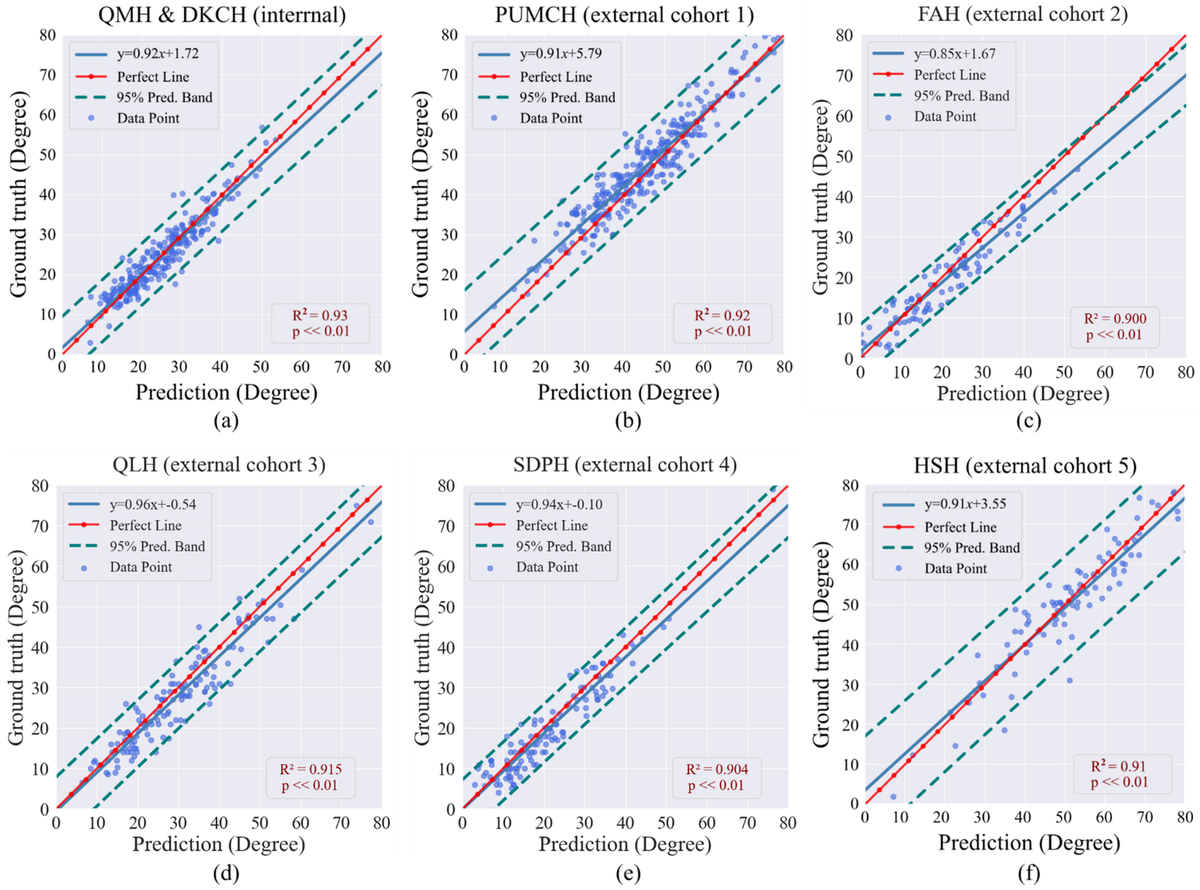
The enhanced model’s linear regression analysis for maximum CA prediction on the internal validation dataset and 5 external validation datasets. The x-axis represents the values predicted by our model, while the y-axis denotes the GT CAs derived from manually annotated landmarks. DKCH: Duchess of Kent Children's Hospital; FAH: First Affiliated Hospital; HSH: Shanghai Huashan Hospital; PUMCH: Peking Union Medical College Hospital; QLH: Qilu Hospital of Shandong University; QMH: Queen Mary hospital; SDPH: Shandong Provincial Hospital.

Linear regression results of enhanced models for maximum CA prediction are shown in Figure 3. In all cohorts, the enhanced model achieved a coefficient of determination (*R*²) exceeding 0.90, peaking at 0.93 on the internal dataset. Specifically, *R*² improved by 0.02 in the internal dataset (enhanced model: 0.93 vs original model: 0.91). Among the independent external datasets, the enhanced model’s *R*² values increased by 0.11 on average relative to the original model, specifically by 0.05 at PUMCH, 0.19 at FAH, 0.10 at QLH, 0.09 at SDPH, and 0.09 at HSH (Figure S2). Bland-Altman analysis was performed to quantify the agreement between predicted and true CAs (Figure S3). Compared to the original model, the enhanced model exhibited narrower 95% LoA intervals across all datasets (QMH&DKCH: 2.24°; PUMCH: 6.16°; FAH: 8.26°; QLH: 9.32°; SDPH: 7.89°; and HSH: 5.93°).

### Disease Severity Grading and Curve Type Identification

Table 3 presents the quantitative performance of the enhanced model in disease severity grading and spinal curve type identification. In the internal validation dataset, the enhanced model achieved an average (macro-average) 3-class sensitivity of 89.97% and a NPV of 94.49%, representing an improvement of 3.85% and 1.74%, respectively, compared to the original model (Table S5 and Figure S4 in Multimedia Appendix 1). Across 5 independent external datasets, the enhanced model attained an average sensitivity of 90.18% and an average NPV of 93.16%, representing substantial improvements of 12.57% and 2.86%, respectively, over the original model (average sensitivity: 77.61% and average NPV: 90.30%).

Regarding spinal curve type classification, the enhanced model slightly improved sensitivity to 99.24% on the internal dataset compared to 98.79% with the original model. However, performance on independent external datasets varied across centers, with both improvements and declines observed (detailed data in Tables S3 and S5 in Multimedia Appendix 1).

**Table 3:** Quantitative results on severity grading and curve type detection using enhanced model.

Evaluation metrics		Severity level					Curve type		
		Normal- mild (0°-20°), coefficient (95% CI)	Moderate (21°-40°), coefficient (95% CI)	Severe (>41°), coefficient (95% CI)	Macro Average	Thoracic, coefficient (95% CI)		Thoracolumbar or lumbar, coefficient (95% CI)	Macro Average
**Internal validation dataset (QMH&DKCH^a^ cohort)**									
	Sensitivity	89.80 (82.23-94.36)	92.62 (87.26-95.83)	87.50 (63.98-96.50)	89.97	98.48 (95.64-99.48)		100 (94.87-100)	99.24
	Specificity	95.15 (90.73-97.52)	89.47 (82.50-93.88)	98.79 (96.49-99.59)	94.47	100 (94.42-100)		97.40 (94.05-98.88)	98.70
	Precision	91.67 (84.41-95.72)	92 (86.54-95.36)	82.35 (58.97-93.81)	88.67	100 (98.07-100)		93.42 (85.51-97.16)	96.71
	NPV^b^	94.01 (89.33-96.72)	90.27 (83.41-94.48)	99.19 (97.08-99.78)	94.49	95.59 (87.81-98.49)		100 (97.99-100)	97.79
	Accuracy	93.16 (89.44-95.63)	91.25 (87.22-94.10)	98.10 (95.63-99.19)	94.17	98.86 (96.70-99.61)		98.10 (95.63-99.19)	98.48
**External validation dataset 1 (PUMCH^c^ cohort)**									
	Sensitivity	100 (51.01-100)	87.78 (79.43-93.04)	86.82 (81.71-90.66)	91.53	97.91 (95.51-99.04)		100 (88.97-100)	98.95
	Specificity	99.03 (97.19-99.67)	87.05 (82.03-90.83)	91.49 (84.10-95.62)	92.53	100 (87.54-100)		99.29 (97.46-99.81)	99.65
	Precision	57.14 (25.05-84.18)	73.15 (64.10-80.61)	95.98 (92.27-97.95)	75.42	100 (98.65-100)		93.94 (80.39-98.32)	96.97
	NPV	100 (98.76-100)	94.66 (90.69-96.99)	74.78 (66.13-81.83)	89.81	81.82 (65.61-91.39)		100 (98.65-100)	90.91
	Accuracy	99.04 (97.23-99.67)	87.26 (83.12-90.50)	88.22 (84.18-91.33)	91.51	98.09 (95.89-99.12)		99.36 (97.71-99.83)	98.73
**External validation dataset 2 (FAH^d^ cohort)**									
	Sensitivity	87.27 (75.98-93.70)	91.11 (79.27-96.49)	100 (43.85-100)	95.56	93.22 (83.82-97.33)		78.57 (60.46-89.79)	85.90
	Specificity	93.75 (83.16-97.85)	87.93 (77.12-94.03)	99 (94.55-99.82)	93.47	93.18 (81.77-97.65)		92 (83.63-96.28)	92.59
	Precision	94.12 (84.08-97.98)	85.42 (72.83-92.75)	75 (30.06-95.44)	80.21	94.83 (85.86-98.23)		78.57 (60.46-89.79)	86.70
	NPV	86.54 (74.73-93.32)	92.73 (82.74-97.14)	100 (96.26-100)	96.36	91.11 (79.27-96.49)		92 (83.63-96.28)	91.56
	Accuracy	90.29 (83.04-94.64)	89.32 (81.88-93.93)	99.03 (94.70-99.83)	94.17	93.20 (86.63-96.67)		88.35 (80.73-93.21)	90.78
**External validation dataset 3 (QLH^e^ cohort)**									
	Sensitivity	83.33 (69.40-91.68)	88.73 (79.31-94.18)	95.24 (77.33-99.15)	91.99	97.56 (87.40-99.57)		96.26 (90.78-98.54)	96.91
	Specificity	97.83 (92.42-99.40)	87.30 (76.89-93.42)	94.69 (88.90-97.54)	91	98.92 (94.16-99.81)		88.89 (71.94-96.15)	93.91
	Precision	94.59 (82.30-98.50)	88.73 (79.31-94.18)	76.92 (57.95-88.97)	82.83	97.56 (87.40-99.57)		97.17 (92.01-99.03)	97.37
	NPV	92.78 (85.85-96.46)	87.30 (76.89-93.42)	99.07 (94.94-99.84)	93.19	98.92 (94.16-99.81)		85.71 (68.51-94.30)	92.32
	Accuracy	93.28 (87.73-96.43)	88.06 (81.48-92.52)	94.78 (89.61-97.45)	91.42	98.51 (94.72-99.59)		94.78 (89.61-97.45)	96.64
**External validation dataset 4 (SDPH^f^ cohort)**									
	Sensitivity	75.31 (64.92-83.41)	80.43 (66.83-89.35)	88.89 (56.50-98.01)	84.66	92.11 (79.20-97.28)		94.19 (87.10-97.49)	93.15
	Specificity	90.91 (80.42-96.05)	76.67 (66.95-84.20)	96.85 (92.18-98.77)	86.76	94.90 (88.61-97.80)		62 (48.15-74.14)	78.45
	Precision	92.42 (83.46-96.72)	63.79 (50.93-74.95)	66.67 (39.06-86.19)	65.23	87.50 (73.89-94.54)		81 (72.22-87.49)	84.25
	NPV	71.43 (59.95-80.68)	88.46 (79.50-93.81)	99.19 (95.57-99.86)	93.38	96.88 (91.21-98.93)		86.11 (71.34-93.92)	91.49
	Accuracy	81.62 (74.27-87.23)	77.94 (70.26-84.09)	96.32 (91.68-98.42)	87.13	94.12 (88.82-96.99)		82.35 (75.08-87.84)	88.24
**External validation dataset 5 (HSH^g^ cohort)**									
	Sensitivity	87.34 (78.24-92.98)	85.25 (74.28-92.04)	88.89 (74.69-95.59)	87.16	87.21 (78.53-92.71)		82.98 (69.86-91.11)	85.09
	Specificity	95.88 (89.87-98.38)	87.83 (80.60-92.61)	96.43 (91.91-98.47)	93.38	98.89 (93.97-99.80)		93.02 (87.27-96.29)	95.96
	Precision	94.52 (86.74-97.85)	78.79 (67.49-86.92)	86.49 (72.02-94.09)	86.60	98.68 (92.92-99.77)		81.25 (68.06-89.81)	89.97
	NPV	90.29 (83.04-94.64)	91.82 (85.18-95.64)	97.12 (92.83-98.88)	93.08	89 (81.37-93.75)		93.75 (88.15-96.80)	91.38
	Accuracy	92.05 (87.09-95.20)	86.93 (81.15-91.13)	94.89 (90.57-97.29)	91.29	93.18 (88.46-96.06)		90.34 (85.08-93.88)	91.76

^a^QMH&DKCH: Queen Mary Hospital and Duchess of Kent Children’s Hospital.

^b^NPV: negative predictive value.

^c^PUMCH: Peking Union Medical College Hospital.

^d^FAH: First Affiliated Hospital, Zhejiang University School of Medicine.

^e^QLH: Qilu Hospital of Shandong University.

^f^SDPH: Shandong Provincial Hospital.

^g^HSH: Shanghai Huashan Hospital.

## Discussion

### Principal Findings

In this multicenter retrospective study, we enhanced and validated SpineHRNet+, a previously single-center validated AI model for automated spine alignment analysis, when applied to data from 5 independent external medical centers. Initial results showed that although SpineHRNet+ performed well within the center where it was originally developed, its accuracy and consistency decreased when applied to external datasets, primarily due to substantial variations in data characteristics across centers. To mitigate this issue, we integrated a novel data transformation technique into the AI model’s training and fine-tuning processes, enabling it to adapt more effectively to differences among various medical centers. Following this approach, the model’s ability to accurately analyze spinal alignment improved significantly across diverse clinical environments.

Previous studies have demonstrated that AI-based approaches can reliably quantify spinal deformities, enhancing clinical assessments of AIS [26,27]. However, such models typically undergo validation using data from single institutions, with limited evaluation of their generalizability across multiple medical institutions [28-30]. Variability across clinical environments, such as differences in equipment, acquisition protocols, patient positioning, and data interpretation practices, can substantially impact the performance of AI models when deployed more broadly [16,17]. To address this challenge, advanced research has explored generative AI techniques to standardize imaging characteristics across datasets by transforming images to match the style of the training data [31,32]. While effective in some cases, these methods typically require large amounts of pixel-level registered data and may introduce image distortions, limiting their clinical applicability in diverse settings [33,34].

In contrast, our study introduced a computationally efficient and clinically applicable data transformation method focusing on standardizing key quantitative characteristics of spinal radiographs across different health care settings, without altering critical anatomical information of spine. This algorithm uses statistical analysis of pixel intensity distributions combined with optimization techniques to achieve unsupervised data transformation, eliminating the need for extensive pixel-level matched data or high computational resources. With a computational complexity of *0*(*n* log *n*), it efficiently processes images in real time (detailed in Section 3.3 in Multimedia Appendix 1). This practicality facilitates real-time processing and broad applicability across clinical settings.

Our findings demonstrate that the enhanced SpineHRNet+ model substantially improved in accuracy and consistency for CA prediction across both internal and external validation cohorts. Specifically, the mean absolute prediction errors were consistently decreased for both internal and independent external datasets, with a particularly notable reduction on external data. This outcome suggests that the proposed method not only improves overall accuracy but also has an even greater impact on cross-center data. In addition, the enhanced model narrowed the *R*² gap between internal and external datasets to less than 0.03, keeping regression slope differences within 0.6° (Figure 3). Bland-Altman analysis further showed that the discrepancy in the LoA interval shrank to within 6.42° (Figure S3 in Multimedia Appendix 1), reflecting improved consistency and stability of AI model performance across varying imaging environments. Given the uneven distribution of disease severity across medical centers (Table 1), where severe cases pose greater analytical challenges than mild cases for CA prediction, the enhanced model’s consistency across datasets further underscores the contribution of our method in improving the robustness of AI models in handling varying disease severity levels.

The improvements in disease severity grading further validate the effectiveness of the proposed method. In the internal validation dataset, the enhanced model achieved a macro-average sensitivity and NPV increase of 3.85% and 1.74%, respectively. In independent external datasets, the enhancements were even more pronounced, with sensitivity improvements ranging from 4.53% to 11.88% and NPV increases between 1.37% and 5.26% (Table S5, Figure S4, and Table S3 in Multimedia Appendix 1). These advancements narrowed the performance gap between internal and external data, further demonstrating the effectiveness and scalability of the proposed method in handling heterogeneous medical imaging data. Notably, this plug-and-play design allows for seamless integration into existing AI pipelines without the need for retraining, with performance enhancements achievable through minimal calibration. It demonstrated the most substantial improvements on external datasets, with prediction mean error reductions 1.4°, and average sensitivity gains 12.57%. External cohorts exhibited distinct severity distributions (eg, PUMCH mean CA 46.6° vs training 25°). This heterogeneity underscores the model’s generalizability, as performance remained robust despite these differences, validating its clinical scalability.

The improved sensitivity and NPV for severity grading suggest enhanced reliability in identifying moderate-to-severe cases that may benefit from early bracing intervention. For instance, in the internal validation cohort, the model correctly identified 92.62% of moderate cases and 87.50% of severe cases, reducing the risk of under-treatment. Confusion matrix analysis (Table 3) further supports the model’s ability to minimize false negatives, which is critical in avoiding missed opportunities for timely intervention. Although some improvements in mean absolute error and *R*² appear numerically modest in the internal dataset, the gains across external datasets were substantial and clinically meaningful. For instance, the enhanced model reduced CA prediction error by up to 2.35° and significantly improved *R*² in external cohorts. These improvements led to enhanced sensitivity in severity grading, directly influencing treatment decisions, such as initiating bracing in borderline cases and improving diagnostic reliability across centers. The mean error of the enhanced model, approximately 4°, is comparable to or even better than the reported inter-observer variability, which ranges from 4.9° to 10°. This finding suggests not only the model's accuracy but also its clinical relevance, highlighting its potential for reliable application in practice.

The lower precision observed in mild classification (eg, 57.14% in PUMCH) reflects a trade-off with sensitivity. The model prioritizes sensitivity to avoid missing true positives, especially in moderate and severe cases, which may lead to increased false positives in mild cases. This is partly due to class imbalance and overlapping radiographic features. In clinical workflows, such predictions should be reviewed by clinicians, particularly for borderline cases, to ensure appropriate management decisions.

The real-time processing capability of our model, with an average inference time of 300 milliseconds per image (excluding approximately 200 milliseconds required for the transformation step), supports seamless integration into clinical workflows. Combined with the transformation step, the total processing time remains under 500 milliseconds, enabling potential deployment within picture archiving and communication system/radiology information system without disrupting existing infrastructure.

### Limitations

Despite its advantages, the proposed data transformation has certain limitations. First, it primarily addresses variability within a single modality (eg, radiographic data) arising from equipment and protocol differences across institutions, but it does not accommodate cross-modality transformations, such as conversions between magnetic resonance imaging, computed tomography, and radiographs. Additionally, as the method focuses solely on adjusting quantitative properties of clinical data without modifying image content, challenges related to image class imbalance, low resolution, or issues associated with suboptimal data quality remain unresolved. BMI data were unavailable for 3 external cohorts, limiting analysis of body habitus effects on radiographic quality. Future research should explore more comprehensive solutions to integrate content-aware enhancements while maintaining the anatomical integrity of medical imaging data, and explore adapting this transformation framework to cross-modality scenarios, such as aligning radiographs with computed tomography or magnetic resonance imaging data. While technically challenging, such extensions could enable unified AI models across imaging modalities, enhancing diagnostic versatility.

### Conclusions

In conclusion, our multicenter study demonstrates that a previously validated AI model for AIS assessment can achieve significantly enhanced performance through the integration of a data transformation method specifically designed to address variations in clinical data across institutions. By improving consistency and accuracy across diverse health care settings in multiple medical centers, our findings highlight the necessity of addressing data variability to ensure the reliable, scalable implementation of AI models in clinical practice. The proposed methodology underscores broader clinical implications, emphasizing the importance of adaptable and robust AI solutions capable of supporting equitable and accurate patient care across diverse health care environments.

## Appendix

Multimedia Appendix 1 Multicenter data collection procedures, inclusion and exclusion criteria, intensity distribution–based data transformation methodology, heterogeneity analysis, and comparative performance evaluation between the enhanced and original models.

## Abbreviations

AI: artificial intelligence
AIS: adolescent idiopathic scoliosis
CA: Cobb angle
CDF: cumulative distribution function
DKCH: Duchess of Kent Children's Hospital
FAH: First Affiliated Hospital, Zhejiang University School of Medicine
GT: ground truth
HSH: Shanghai Huashan Hospital
IRB: Institutional Review Board
LoA: limits of agreement
NPV: negative predictive value
PUMCH: Peking Union Medical College Hospital
QLH: Qilu Hospital of Shandong University
QMH: Queen Mary Hospital
SDPH: Shandong Provincial Hospital

## Data Availability

The test data is available from the corresponding authors on a reasonable request and under the related institution policy.
