# Supplementary material for: Scalable and Robust Artificial Intelligence for Spine Alignment Assessment: Multicenter Study Enabled by Real-Time Data Transformation
Source: J Med Internet Res. 2026 Mar 20;28:e78396. doi: 10.2196/78396 (PMC13049401; doi:10.2196/78396)
Supplement: Multimedia Appendix 1 [file jmir_v28i1e78396_app1.docx]

Supplementary Materials

**Table of Contents**

[**1. Data Collection** 1](#_Toc213437765)

[**1.1 Data collection in QMH and DKCH** 1](#_Toc213437766)

[**1.2 Data collection in PUMCH** 3](#_Toc213437767)

[**1.3 Data collection in FAH** 4](#_Toc213437768)

[**1.4 Data collection in QLH** 5](#_Toc213437769)

[**1.5 Data collection in SDPH** 7](#_Toc213437770)

[**1.6 Data collection in HSH** 8](#_Toc213437771)

[**2. Inclusion and exclusion criteria** 12](#_Toc213437772)

[**2.1 Inclusion criteria** 12](#_Toc213437773)

[**2.1 Exclusion criteria** 12](#_Toc213437774)

[**3. Data heterogeneity** 14](#_Toc213437775)

[**3.1 Intensity distribution-based data transform algorithm** 14](#_Toc213437776)

[**3.2 Visual illustration of data transformation results** 14](#_Toc213437777)

[**3.3 Complexity Analysis of the Data Transformation Algorithm** 17](#_Toc213437778)

[**3.4 Quantitative evaluation of data heterogeneity** 18](#_Toc213437779)

[**4. Performance comparison between the enhanced model and original model** 20](#_Toc213437780)

[**4.1 Performance analysis on Cobb angle prediction** 20](#_Toc213437781)

[**4.2 Performance analysis on disease severity grading.** 24](#_Toc213437782)

# **1. Data Collection**

This multi-centre study involved data collection from two local hospitals in Hong Kong and five leading hospitals in Mainland China. The local hospitals include Queen Mary Hospital (**QMH**) and Duchess of Kent Children's Hospital (**DKCH**), while the Mainland hospitals consist of 1) Peking Union Medical College Hospital, Beijing, China (**PUMCH** cohort), 2) First Affiliated Hospital, Zhejiang University School of Medicine, Hangzhou, China (**FAH** cohort), 3) Qilu Hospital of Shandong University, Jinan, China (**QLH** cohort), 4) Shandong Provincial Hospital, Jinan, China (**SDPH** cohort), and 5) Huashan Hospital, Shanghai, China (**HSH** cohort). Variations in data collection protocols across these hospitals have led to issues of data heterogeneity among the different centres.

## **1.1 Data collection in QMH and DKCH**

At the Queen Mary Hospital and Duchess of Kent Children’s Hospital (QMH&DKCH), the Radiology Department conducts comprehensive evaluations of patients diagnosed with adolescent idiopathic scoliosis (AIS) through full-length spine radiographs. The imaging is performed using biplanar stereoradiography or EOS imaging (Manufacturer: EOS® system, Model: EOS3.4), ensuring high-resolution and reliable diagnostic images.

**Pre-Imaging Preparation**

Prior to the radiographic procedure, patients are instructed to remove all metallic objects, including jewelry, belts, and clothing with metallic components. This precautionary measure is essential to prevent image artifacts that could compromise diagnostic clarity. Additionally, patients receive a thorough briefing about the procedure to alleviate any apprehensions and ensure cooperative participation during image acquisition.

**Patient Positioning and Imaging Views**

The EOS system facilitates biplane imaging, capturing simultaneous posteroanterior (PA) and lateral views. This dual-view approach enhances the accuracy of spinal curvature assessments by providing comprehensive sagittal and coronal plane images. For obtaining PA and lateral views of the spine, patients are positioned upright whilst using the EOS imaging system. They stand on the designated pedal with their backs firmly aligned against the imaging panel. Feet are placed shoulder-width apart, and arms are relaxed by their sides. Patients maintain a natural posture with eyes fixed straight ahead, ensuring that the sagittal planes of the head, neck, chest, and abdomen remain horizontally aligned with the machine's central axis.

**Imaging Parameters**

The imaging settings at QMH&DKCH are meticulously calibrated to optimize image quality and diagnostic utility:

- **Kilovolt Peak (KVP):** 75.0 kV
- **Image and Fluoroscopy Area Dose Product:** 143.29 mGy·cm²
- **Exposure time:** 100 ms
- **Pixel Spacing:** 0.179363 mm × 0.179363 mm
- **Pixel Representation:** Unsigned integers (0)
- **Imager Pixel Spacing:** 0.254 mm × 0.254 mm

**Quality Assurance and Safety**

Upon acquisition, radiologists promptly assess each image for clarity and completeness. Any images that do not meet the quality standards are retaken to ensure the integrity of the diagnostic data. All imaging procedures comply strictly with established radiation safety protocols, minimizing patient exposure while maintaining optimal image quality.

**Documentation and Data Integrity**

Comprehensive records of each imaging session are meticulously maintained within the hospital’s digital medical records system. This includes detailed metadata extracted from DICOM headers, such as Study Instance UID and Series Instance UID, ensuring seamless integration with the hospital’s medical records system. These records support data integrity and facilitate efficient retrieval and analysis of patient information.

**Customization and Adaptability**

While utilizing the EOS imaging system, QMH&DKCH has tailored specific imaging settings to address the unique characteristics and clinical needs of its patient population. These adjustments include customized exposure settings and pixel spacing calibrations, enhancing the system’s flexibility and effectiveness. This customization underscores the robustness of our algorithm in managing diverse and heterogeneous data across various clinical environments.

## **1.2 Data collection in PUMCH**

At Peking Union Medical College Hospital (PUMCH), the Department of Radiology conducts comprehensive evaluations of patients diagnosed with spinal deformities through full-length spine radiographs. The imaging is carried out using the **Philips Medical Systems DigitalDiagnost system**, ensuring high-resolution and reliable diagnostic images.

**Pre-Imaging Preparation:** Prior to the radiographic procedure, patients are advised to eliminate all metallic items, including accessories and garments with metal parts, to avoid potential distortions or artifacts in the images.

**Patient Positioning:**

Patients are instructed to stand on the designated pedal while placing their backs firmly against the imaging panel. They maintain a natural posture with feet shoulder-width apart, arms relaxed by their sides, and gaze fixed forward. This alignment guarantees that the sagittal planes of the head, neck, chest, and abdomen remain horizontally aligned with the machine's central axis.

**Imaging Parameters:** The imaging settings at PUMCH are meticulously calibrated to optimize image quality and diagnostic utility:

- **Pixel Spacing:** 0.13598 mm × 0.13598 mm
- **Pixel Representation:** Unsigned integers (0)
- **Spatial Resolution:** 0.143 mm
- **Image and Fluoroscopy Area Dose Product:** 13.285 mGy·cm²
- **Imager Pixel Spacing:** 0.143 mm × 0.143 mm

**Quality Assurance and Safety:** Upon acquisition, radiologists promptly assess each image for clarity and completeness. Any images that do not meet the quality standards are promptly retaken to ensure the integrity of the diagnostic data. All imaging procedures comply strictly with established radiation safety protocols, minimizing exposure while maintaining optimal image quality.

**Documentation and Data Integrity:** Detailed records of each imaging session are maintained meticulously, ensuring seamless integration with the hospital’s medical records system. This rigorous documentation supports data integrity and facilitates efficient retrieval and analysis of patient information.

**Customization and Adaptability:** While utilizing the **Philips DigitalDiagnost system**, PUMCH has tailored specific imaging settings to address the unique characteristics and clinical needs of its patient population. These adjustments enhance the system’s flexibility and effectiveness, demonstrating the robustness of our algorithm in managing diverse and heterogeneous data across various clinical environments.

## **1.3 Data collection in FAH**

At First Affiliated Hospital, Zhejiang University School of Medicine (FAH), the Radiology Department employs a meticulous data collection protocol to ensure the accuracy and consistency of spinal imaging studies. Utilizing the **GE Healthcare Discovery XR656 system**, all patients presenting with suspected lumbar spine abnormalities undergo standardized radiographic evaluations. This protocol outlines the procedural steps, equipment specifications, patient preparation, imaging parameters, and quality assurance measures integral to the data collection process.

**Pre-Imaging Preparation**

Prior to the commencement of the radiographic procedure, patients are instructed to remove any metallic objects, including jewelry, belts, and clothing with metallic fastenings. This precautionary measure is essential to prevent image artifacts that could compromise diagnostic clarity. Additionally, patients are briefed on the procedure to alleviate any apprehensions and ensure cooperative participation during image acquisition.

**Patient Positioning and Imaging Views**

For obtaining PA views of the spine, patients are positioned upright against the imaging panel. They stand on the designated pedal, ensuring their backs are firmly aligned with the panel. The feet are placed shoulder-width apart, and arms are relaxed by the sides. Patients are instructed to maintain a natural posture with eyes fixed straight ahead. This alignment guarantees that the sagittal planes of the head, neck, chest, and abdomen remain horizontally aligned with the machine's central axis, facilitating accurate lateralization of spinal structures.

**Imaging Equipment and Settings**

The GE Healthcare Discovery XR656 system is configured with the following specifications to optimize image quality and diagnostic utility:

- **KVP (Kilovolt Peak):** 80.0 kV
- **Exposure Time:** 161 ms
- **X-Ray Tube Current:** 500 mA
- **Exposure:** 81 mAs
- **Image and Fluoroscopy Area Dose Product:** 27.266155 mGy·cm²
- **Imager Pixel Spacing:** 0.187142 mm × 0.187142 mm

**Quality Assurance and Radiation Safety**

Each radiographic image is promptly reviewed by a qualified radiologist to ensure clarity, completeness, and diagnostic adequacy. Images that exhibit any form of distortion, underexposure, or other quality issues are promptly retaken to maintain the integrity of the diagnostic data. FAH adheres strictly to established radiation safety protocols, minimizing patient exposure by optimizing imaging parameters and employing appropriate shielding techniques.

**Documentation and Data Management**

Comprehensive records of each imaging session are meticulously maintained within the hospital’s digital medical records system. This includes detailed metadata extracted from DICOM headers, such as Study Instance UID, Series Instance UID, Patient ID, and Procedure Step IDs. These records facilitate efficient data retrieval, longitudinal patient tracking, and integration with broader clinical workflows.

**Customization and Adaptability**

While the **GE Healthcare Discovery XR656 system** serves as the primary imaging platform, FAH has implemented tailored imaging settings to address the specific clinical requirements of its patient population. This customization enhances the flexibility and effectiveness of the imaging protocol, ensuring optimal diagnostic outcomes across diverse clinical scenarios.

## **1.4 Data collection in QLH**

At Jinan Qilu Hospital of Shandong University (QLH), the Department of Radiology conducts comprehensive evaluations of patients diagnosed with AIS using full-length spine radiographs. Data collection is performed using a variety of imaging devices, including the **KODAK DIRECTVIEW DR 7500**, **Canon Digital Radiography**, **CARESTREAM DRX-EVOLUTION**, and **EOS imaging systems**, ensuring high-resolution and reliable diagnostic images.

**Pre-Imaging Preparation**

Prior to the radiographic procedure, patients are instructed to remove all outer clothing, shoes, and metal objects to prevent image artifacts. This includes eliminating jewelry, belts, and garments with metallic components. Additionally, patients receive a thorough briefing about the procedure to alleviate any concerns and ensure their cooperation during image acquisition.

**Patient Positioning and Imaging Views**

Patients are guided to stand naturally without exertion in a marked position. They should position their feet slightly apart with aligned heels and toes, and maintain naturally extended knees and hips. The body must remain upright, with the head in a neutral position and eyes directed forward. Arms are positioned forward with elbows bent, and hands are placed at cheek level without touching the body. For patients with leg length discrepancies, heel lifts or insoles are provided to ensure proper alignment. Patients who wear orthotics are advised to continue wearing them, except for those undergoing orthopedic treatment, who remove their orthotics a day prior to the procedure. Individuals unable to stand unaided are assisted to maintain balance without altering their posture. This meticulous positioning protocol ensures consistent and accurate depiction of spinal structures across all imaging sessions.

**Imaging Parameters**

JSTH utilizes multiple imaging devices, each with its specific configuration to cater to diverse clinical needs. While specific imaging parameters may vary depending on the device used, standardized protocols are implemented to maintain image quality and diagnostic reliability.

All imaging procedures adhere strictly to established radiation safety guidelines to minimize patient exposure while maintaining optimal image quality. Radiologists promptly review each acquired image for clarity and completeness. Any images that do not meet quality standards are retaken to ensure the integrity of the diagnostic data. This rigorous quality assurance process guarantees that all radiographs are suitable for accurate clinical assessment and research purposes.

**Documentation and Data Integrity**

Comprehensive records of each imaging session are meticulously maintained within the hospital’s digital medical records system. This includes detailed metadata extracted from DICOM headers, such as Study Instance UID and Series Instance UID, ensuring seamless integration with the hospital’s medical records infrastructure. These records support data integrity, facilitate efficient retrieval, and enable robust analysis of patient information across different cohorts and studies.

**Customization and Adaptability**

QLH employs a diverse array of imaging devices tailored to meet the unique clinical requirements of its patient population. The use of multiple systems, including KODAK, Canon, CARESTREAM, and EOS, allows for flexibility in imaging protocols and enhances the robustness of the study by incorporating varied imaging techniques. This heterogeneity in data collection underscores the algorithm’s capability to handle diverse data sources, thereby improving its generalizability and applicability across different clinical environments. Such customization ensures that the imaging protocols remain effective and relevant, accommodating the specific needs of each patient and clinical scenario.

## **1.5 Data collection in SDPH**

At Shandong Provincial Hospital, Jinan, the radiologists conduct comprehensive evaluations of patients diagnosed with spinal deformities using full-length spine radiographs. Imaging is performed with the **EOS Imaging System (EOS® Imaging Inc.)**, which captures simultaneous biplanar (PA and lateral) radiographs, ensuring high-resolution and reliable diagnostic images.

**Pre-Imaging Preparation**

Prior to the radiographic procedure, patients are required to remove all metal objects, including jewellery and clothing with metallic components, to prevent image artifacts. Additionally, patients receive a thorough briefing about the procedure to alleviate any apprehensions and ensure their cooperation during image acquisition.

**Patient Positioning and Biplanar Imaging**

Using the EOS system's capability to acquire biplanar images simultaneously, patients are positioned to optimize spinal alignment and image quality:

- **Standing Position:** Patients stand upright in a natural posture on the designated pedal, ensuring their backs are firmly aligned against the imaging panels. Feet are placed shoulder-width apart, and arms are relaxed by their sides. Eyes are directed straight ahead to maintain head and neck alignment.
- **Biplanar Acquisition:** The EOS system simultaneously captures both AP and lateral views of the spine. This dual-view approach eliminates the need for separate imaging sessions for AP and lateral views, reducing patient exposure to radiation and minimizing positional discrepancies between images. The simultaneous acquisition ensures that the sagittal planes of the head, neck, chest, and abdomen remain horizontally aligned with the machine's central axis, and the body's median line coincides with the machine's median line.

**Imaging Parameters**

While specific imaging parameters vary, the EOS system at Shandong Provincial Hospital is calibrated to optimize image quality and diagnostic utility. Key parameters include automatic exposure control to ensure consistent image brightness and contrast across different patients and imaging sessions. The system's dual-plane imaging capability facilitates simultaneous acquisition of sagittal and coronal views, enhancing the accuracy of spinal assessments.

**Quality Assurance and Safety**

Radiologists promptly review each acquired image for clarity and completeness. Any images that do not meet quality standards are retaken to ensure the integrity of the diagnostic data. All imaging procedures adhere strictly to established radiation safety regulations, minimizing patient exposure while maintaining optimal image quality.

**Customization and Adaptability**

While utilizing the EOS Imaging System, Shandong Provincial Hospital has tailored specific imaging settings to address the unique characteristics and clinical needs of its patient population. These adjustments enhance the system’s flexibility and effectiveness, demonstrating the robustness of our algorithm in managing diverse and heterogeneous data across various clinical environments.

## **1.6 Data collection in HSH**

At Huashan Hospital, the Radiology Department adheres to a rigorous data collection protocol to ensure the precision and consistency of spinal imaging studies. Utilizing the **GE Healthcare Optima XR646 HD system**, all patients requiring lumbar spine evaluations undergo standardized radiographic examinations. This protocol delineates the procedural steps, equipment specifications, patient preparation, imaging parameters, and quality assurance measures essential for effective data collection.

**Pre-Imaging Preparation**

Before initiating the radiographic procedure, patients are instructed to remove all metallic objects, including jewelry, belts, and clothing with metallic components. This step is crucial to prevent image artifacts that may compromise diagnostic accuracy. Additionally, patients receive a comprehensive briefing about the procedure to ensure their understanding and cooperation, thereby facilitating a smooth and efficient imaging session.

**Patient Positioning and Imaging Views**

For acquiring AP views of the spine, patients are positioned upright against the imaging panel. They stand on the designated pedal, ensuring their backs are firmly aligned with the panel. Feet are placed shoulder-width apart, and arms are relaxed by their sides. Patients are instructed to maintain a natural posture with eyes fixed straight ahead. This alignment ensures that the sagittal planes of the head, neck, chest, and abdomen remain horizontally aligned with the machine's central axis, promoting accurate depiction of spinal structures.

**Imaging Equipment and Settings**

The GE Healthcare Optima XR646 HD system is configured with the following specifications to optimize image quality and diagnostic utility:

**Technical Parameters:**

- **KVP (Kilovolt Peak):** 100.0 kV
- **Exposure:** 15 mAs
- **Image Dose:** 0.431294 mGy
- **Pixel Spacing:** 0.1697 mm × 0.1697 mm
- **Imager** **Pixel Spacing:** 0.2 mm × 0.2 mm

**Quality Assurance and Radiation Safety**

Each radiographic image undergoes immediate evaluation by a qualified radiologist to ensure clarity, completeness, and diagnostic adequacy. Images displaying any form of distortion, underexposure, or other quality issues are promptly retaken to maintain the integrity of the diagnostic data. Huashan Hospital strictly adheres to established radiation safety protocols, minimizing patient exposure by optimizing imaging parameters and employing appropriate shielding techniques.

**Documentation and Data Management**

Comprehensive records of each imaging session are meticulously maintained within the hospital’s digital medical records system. This includes detailed metadata extracted from DICOM headers, such as Study Instance UID, Series Instance UID, Patient ID, and Procedure Step IDs. These records facilitate efficient data retrieval, longitudinal patient tracking, and seamless integration with broader clinical workflows.

**Customization and Adaptability**

While the GE Healthcare Optima XR646 HD system serves as the primary imaging platform, Huashan Hospital has implemented tailored imaging settings to address the specific clinical requirements of its patient population. This customization enhances the flexibility and effectiveness of the imaging protocol, ensuring optimal diagnostic outcomes across diverse clinical scenarios.

**Table S1.**  Experimental settings of different radiographic imaging devices in different medical centres.

| **Medical Center & Imaging Device** | **KVP (kV)** | **Exposure Time (ms)** | **Exposure**  **(mAs)** | **Pixel Spacing (mm)** | **Imager Pixel Spacing (mm)** | **Image Dose**  **(mGy)** | **Area Dose Product**  **(mGy·cm²)** |
| --- | --- | --- | --- | --- | --- | --- | --- |
| **QMH&DKCH**  (EOSedge^TM^) | 75 | 100 |  | 0.179363× 0.179363 | 0.254×0.254 | - | 143.29 |
| **PUMCH**  (Philips DigitalDiagnost) | - | - | - | 0.13598× 0.13598 | 0.143×0.143 | - | 13.285 |
| **FAH**  (GE Healthcare Discovery XR656) | 80 | 161 | 81 | - | 0.187142× 0.187142 | - | 27.266155 |
| **QLH**  (KODAK DIRECTVIEW DR 7500;  Canon DR; CARESTREAM DRX-EVOLUTION;  EOS Imaging System) | - | - |  | - | - | - | - |
| **SDPH**  (EOS Imaging System) | - | - |  | - | - | - | - |
| **HSH**  (GE Healthcare Optima XR646 HD) | 100 | - | 15 | 0.1697× 0.1697 | 0.2×0.2 | 0.431294 | - |

* **KVP:** Kilovolt Peak

# **2. Inclusion and exclusion criteria**

## **2.1 Inclusion criteria**

1. **Age and Diagnosis**: Male and female individuals aged between 10 and 18 years old diagnosed with adolescent idiopathic scoliosis (AIS), experiencing low back pain, or presenting with degenerative spinal deformities.
2. **Informed Consent**: Participants, or their legal guardians if participants are under 18 years old, must be able to provide written informed consent, available in either English or Chinese.
3. **Understanding of Study**: Participants must comprehend the objectives and scope of the study.

## **2.1 Exclusion criteria**

1. **Psychological Disorders**: Individuals diagnosed with or exhibiting signs of psychological disorders that could affect their ability to comply with study protocols.
2. **Neurological Conditions**: Patients with diagnosed systemic neurological disorders that may impair mobility, such as previous cerebrovascular accidents, Parkinson’s disease, or myopathies.
3. **Musculoskeletal Diseases**: Exclusion applies to those with the following conditions:

- Congenital spinal deformities
- McCune-Albright syndrome
- Early-onset scoliosis
- History of spinal surgery or spinal instrumentation
- Traumatic injuries that could affect posture or movement

1. **Oncological Diseases**: Individuals diagnosed with any form of cancer.
2. **Systemic Diseases**: Individuals with other systemic illnesses not specified above.
3. **Consent Inability**: Patients unable to complete the consent process.

**Table S2.** Deformity severity assessment standard and clinical management.

| **Max Cobb angle (CA)** | **Severity** | **Clinical Management** |
| --- | --- | --- |
| CA < 20° | Normal-mild | No intervention required. For the skeletally immature, regular follow-up is required every 4-6 months to identify curve progression early in which bracing may be recommended. |
| 20° ≤ CA < 40° | Moderate | Patients are suggested for bracing to prevent curve progression. No intervention may be required at the end of growth. Scoliosis-specific exercises may also be prescribed. |
| CA ≥ 40° | Severe | These severe curves have risk of adulthood progression. Surgical intervention may be required in the form of vertebral body tethering (skeletally immature only) or curve correction and spinal fusion. |

# **3. Data heterogeneity**

Due to variability in radiograph acquisition equipment, standards, and parameter settings across different medical centres, images often exhibit significant differences in brightness, contrast, exposure, etc. These differences are a primary source of data heterogeneity in multi-centre studies. To better characterize this heterogeneity, we analysed the *pixel intensity distributions* across radiographs from all participating centres and identified substantial inter-centre variability. This variability frequently leads to reduced performance when AI models trained at one centre are directly applied to datasets from other centres.

## **3.1 Intensity distribution-based data transform algorithm**

To address these challenges, we developed a novel data transformation method based on intensity distribution of the radiograph. This method precisely adjusts the intensity values of each pixel to closely match the intensity distribution of a selected reference image (i.e., target image). Notably, this technique is computationally efficient, capable of processing a single radiograph (with a spatial resolution of approximately 800×400) within roughly 200 milliseconds. A detailed description of this algorithm is provided in Algorithm S1.

## **3.2 Visual illustration of data transformation results**

Figure S1 visually demonstrates the efficacy of the proposed intensity distribution–based data transformation method. As depicted, the first row (red panel) displays original radiographs collected from five external medical centres, along with their corresponding pixel intensity distributions beneath each image. The first column (green panel) presents radiographs sourced from QMH&DKCH, showcasing three distinct images. The image grid (blue panel) demonstrates the transformed radiographs obtained through the intensity distribution-based data transformation algorithm applied to radiographs from QMH&DKCH, and the intensity distributions of each transformed images are presented below each transformed image. The transformed radiographs exhibit pixel intensity distributions closely matching those from the external centres, underscoring the effectiveness of the proposed data transformation approach in mitigating data heterogeneity.


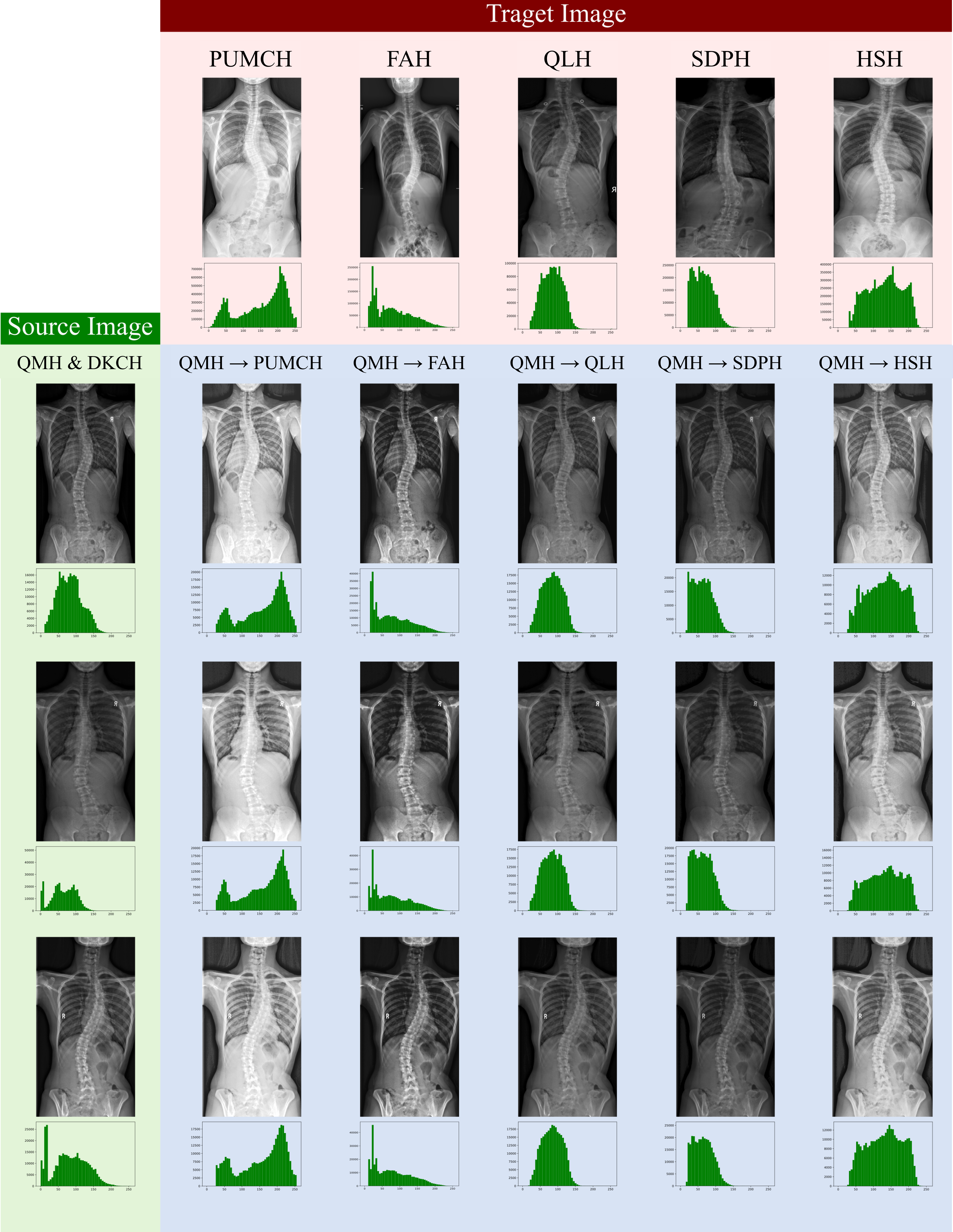


**Figure S1.** Visual results of intensity distribution-based data transform algorithm. The first row presents the sample radiographs obtained from 5 different external medical centres (PUMCH, FAH, QLH, SDPH, and HSH), while the first column exhibits the radiographs obtained from QMH&DKCH. The intensity distribution of each radiograph is presented under the corresponding image.

| **Algorithm 1: intensity distribution-based data transform algorithm** | | |
| --- | --- | --- |
|  | **Inputs:** source image $I^{S}$; target image $I^{T}$. | |
|  | **Outputs:** transformed image $\hat{I}$. | |
| 1: | $I_{flat}^{S}$, $I_{flat}^{T}$← Flatten($I^{S}$), Flatten($I^{T}$) | |
| 2: | $\mathcal{H}^{S}$← [], $\mathcal{H}^{T}$← [] # calculate intensity histogram | |
| 3: | **for each** *p* **in** Length($I_{flat}^{S}$) **do** | |
| 4: |  | $\mathcal{H}^{S}$[$I_{flat}^{S}$ [*p*]] += 1 |
| 5: | **End** | |
| 6: | **for each** *p* **in** Length($I_{flat}^{T}$) **do** | |
| 7: |  | $\mathcal{H}^{T}$[$I_{flat}^{T}$ [*p*]] += 1 |
| 8: | **End** | |
| 9: | $\mathcal{F}^{S}$← [], $\mathcal{F}^{T}$← [] # calculate intensity CDF | |
| 10: | count ← 0 | |
| 11: | **for each** *k* **in** Length($\mathcal{H}^{S}$) **do** | |
| 12: |  | count ← count + $\mathcal{H}^{S}$[*k*] |
| 13: |  | $\mathcal{F}^{S}$ ← Append($\mathcal{F}^{S}$, count / Sum($\mathcal{H}^{S}$)) |
| 14: | **End** | |
| 15: | count ← 0 | |
| 16: | **for each** *k* **in** Length($\mathcal{H}^{S}$) **do** | |
| 17: |  | count ← count + $\mathcal{H}^{T}$[*k*] |
| 18: |  | $\mathcal{F}^{T}$ ← Append($\mathcal{F}^{T}$, count / Sum($\mathcal{H}^{T}$)) |
| 19: | **End** | |
| 20: | **for each** *i* **in** Length($\mathcal{H}^{S}$) **do** # intensity adjustment | |
| 21: |  | $s_{i}$ ← $\mathcal{F}^{S}$[*i*] |
| 22: |  | *j* ← $\underset{t_{j}\boldsymbol{\in}\mathcal{F}^{T}}{\mathbf{argmin}} \left\vert t_{j}\boldsymbol{-}s_{i} \right\vert$ # Optimization |
| 23: |  | $I_{flat}^{S}$[$I_{flat}^{S}$=*i*] ← *j* |
| 24: | **end** | |
| 25: | $\hat{I}$ ← Reshape($I_{flat}^{S}$, Shape($I^{S}$)) | |
| 26: | **return** $\hat{I}$ | |

**Algorithm S1.** The proposed intensity distribution-based data transform algorithm for heterogeneous data simulation.

## **3.3 Complexity Analysis of the Data Transformation Algorithm**

To address the variations in intensity distribution among radiographs from different medical centres, this study introduces a data transformation algorithm based on intensity distribution analysis. The algorithm adjusts the pixel intensity values of source images to align closely with the intensity distribution of target images. This process enhances the robustness and accuracy of deep learning models when applied to heterogeneous datasets. The following section offers an analysis of the computational complexity of the proposed algorithm, as derived from the accompanying pseudocode in Algorithm S1.

Let *n* denote the total number of pixels under consideration. The computational complexity can be analysed in four primary steps:

1. **Flattening the Input Images:**

The process of flattening both the source and target images (line 1 of the algorithm) transforms each *M*×*N* image into a one-dimensional array of length *n*. This operation is performed once for each image and requires $O\left( n \right)$ time.

1. **Histogram Computation (Lines 2–8):**

Computing the intensity histograms $\mathcal{H}^{S}$ and $\mathcal{H}^{T}$ involves a single pass over all pixel intensities of the source and target images, respectively to finding unique values and their counts. Each pass is $O\left( n\log n \right)$. Thus, the total computational cost for histogram generation is $O\left( n\log n \right)$.

1. **Cumulative Distribution Function (CDF) Computation (Lines 9–19):**

Once histograms are obtained, the CDFs $\mathcal{F}^{S}$ and $\mathcal{F}^{T}$ are computed. This requires a single pass over all the intensity levels for each histogram. The cumulative summation of histogram counts is an $O\left( n \right)$ operation, since each pixel’s contribution to the distribution is processed once.

1. **Intensity Adjustment (Lines 20–24):**

The final intensity remapping involves a single pass over the pixel intensities to interpolate and assign new intensity values based on the computed CDFs. This interpolation step is an $O\left( n \right)$ operation.

**Overall Complexity:**

Combining the complexities of all four stages, the dominant term is $O\left( n\log n \right)$ (due to the histogram computation step). Consequently, the overall computational complexity of the proposed data transformation algorithm is $O\left( n\log n \right)$.

## **3.4 Quantitative evaluation of data heterogeneity**

To quantitatively assess data heterogeneity across radiographs from different medical centres, we introduced two quantitative metrics that capture the essential characteristics of the pixel intensity distribution and overall brightness levels of the images. The first metric, **Histogram Spread (HS)**, evaluates the dispersion of pixel intensities within an image. It is calculated based on the interquartile range (IQR) of the pixel intensity histogram, normalized by the possible range of pixel values:

$$HS=\frac{(Q_{3}-Q_{1})}{I_{max}-I_{min}}, (1)$$

where $Q_{1}$ and $Q_{3}$ denote the 1^st^ and 3^rd^ quartile of the intensity histogram, respectively. $I_{max}$ and $I_{min}$ represent the maximum and minimum value of the image, respectively. A higher HS value indicates greater dispersion and heterogeneity in pixel intensity distribution across images.

The second metric is **Brightness**, reflecting the overall luminance level within each radiograph. It is defined as the mean pixel intensity across the entire image. Mathematically, brightness ($B$) is computed as:

$$B=\frac{1}{N}\sum_{i=1}^{N} p_{i}, (2)$$

where $p_{i}$ represents the intensity value of the $i^{th}$ pixel and $N$ is the total number of pixels in the image.

These two metrics collectively enable a quantitative analysis of data heterogeneity, facilitating objective evaluation and comparison across datasets from multiple medical centres.

**Table S3.** Quantitative assessment of data heterogeneity before and after data transformation across different medical centres.

|  | PUMCH | Before data transformation | | After data transformation | |
| --- | --- | --- | --- | --- | --- |
|  |  | QMH&DKCH | Abs. Diff. | QMH&DKCH | Abs. Diff. |
| HS (10^-3^) | 1.94 | 3.18 | 1.24 | 1.96 | 0.02 |
| Brightness | 111.85 | 106.98 | 4.87 | 112.18 | 0.33 |
|  | | | | | |
|  | FAH | Before data transformation | | After data transformation | |
|  |  | QMH&DKCH | Abs. Diff. | QMH&DKCH | Abs. Diff. |
| HS (10^-3^) | 2.17 | 3.18 | 1.01 | 2.22 | 0.05 |
| Brightness | 88.61 | 106.98 | 18.37 | 88.5 | 0.11 |
|  | | | | | |
|  | QLH | Before data transformation | | After data transformation | |
|  |  | QMH&DKCH | Abs. Diff. | QMH&DKCH | Abs. Diff. |
| HS (10^-3^) | 2.53 | 3.18 | 0.65 | 2.61 | 0.08 |
| Brightness | 121.8 | 106.98 | 14.82 | 120.97 | 0.83 |
|  | | | | | |
|  | SDPH | Before data transformation | | After data transformation | |
|  |  | QMH&DKCH | Abs. Diff. | QMH&DKCH | Abs. Diff. |
| HS (10^-3^) | 2.17 | 3.18 | 1.01 | 2.23 | 0.06 |
| Brightness | 89.14 | 106.98 | 17.84 | 90.05 | 0.91 |
|  | | | | | |
|  | HSH | Before data transformation | | After data transformation | |
|  |  | QMH&DKCH | Abs. Diff. | QMH&DKCH | Abs. Diff. |
| HS (10^-3^) | 2.30 | 3.18 | 0.88 | 2.30 | 0.00 |
| Brightness | 79.95 | 106.98 | 27.03 | 80.14 | 0.19 |
| **Average** | | | | | |
|  | Average (over all 5 external datasets) | Before data transformation | | After data transformation | |
|  |  | QMH&DKCH | Abs. Diff. | QMH&DKCH | Abs. Diff. |
| HS (10^-3^) | **2.22** | **3.18** | **0.96** | **2.26** | **0.04** |
| Brightness | **98.27** | **106.98** | **8.71** | **98.37** | **0.47** |

Abbreviation: **HS**: Histogram Spread; **Abs. Diff.**: Absolute Difference.

Table S3 summarizes the histogram spread (HS) and brightness values for radiographs collected from five external centres compared with the internal cohort (QMH&DKCH), both before and after applying the intensity distribution-based data transformation. Absolute differences (Abs. Diff.) quantify the extent of heterogeneity relative to the internal dataset. Data transformation substantially reduced discrepancies in both pixel distribution characteristics (HS) and overall brightness levels across centres.

# **4. Performance comparison between the enhanced model and original model**

To evaluate the effectiveness of the proposed data transformation algorithm in improving the training of AI-based models, we compared the performance of the *enhanced* and *original* models on clinically relevant tasks, including Cobb angle prediction and disease severity grading.

## **4.1 Performance analysis on Cobb angle prediction**

First, we compared the performance of the enhanced and original models in predicting Cobb angles. Figure S2 presents the linear regression analysis for maximum Cobb angle prediction on the internal validation dataset and five external validation datasets. We further conduct a residual analysis of the predicted Cobb angles versus the GT. Key quantitative metrics, including the 25th percentile (Q1), median, 75th percentile (Q3), interquartile range (IQR), mean, and standard deviation (SD) for the box plots, are detailed in Table S4.

The results of mean and variance analysis across different datasets indicate that the enhanced model produces prediction errors that are closer to zero, with a reduced variance compared to the original model, for both internal and external datasets. This suggests that the enhanced model achieves higher prediction accuracy and better alignment with GT values. Furthermore, the IQR comparison reveals that the prediction errors of the enhanced model are more concentrated, demonstrating improved model stability and consistency in Cobb angle prediction across both internal and external datasets.


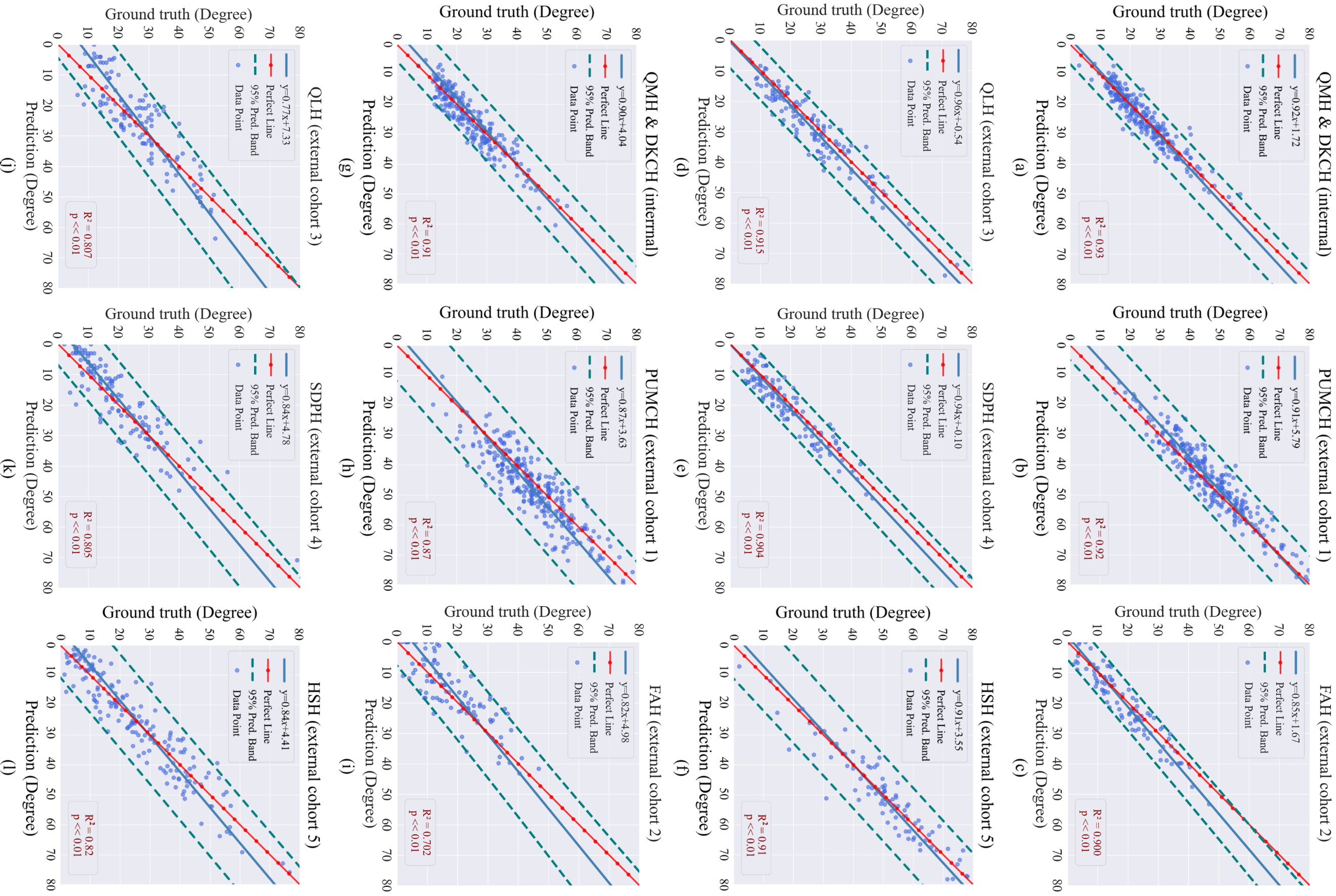


**Figure S2.** Linear regression analysis for maximum Cobb angle prediction on the internal validation dataset and five external validation datasets. The x-axis represents the values predicted by our model, while the y-axis denotes to the GT Cobb angles derived from manually annotated landmarks. Subfigure (a) – (f) illustrate the *enhanced* model’s linear regression results, and subfigure (g) – (l) show those of the *original* model.

**Table S4**. The key parameters in residual of the predicted Cobb angles versus the GT.

| Medical centers | | **QMH & DKCH** | **PUMCH** | **FAH** | **QLH** | **SDPH** | **HSH** |
| --- | --- | --- | --- | --- | --- | --- | --- |
| Thoracic  (original) | Q1 | -0.27 | -0.63 | -6.52 | -7.50 | -5.99 | 0.27 |
|  | Median | 1.83 | 2.04 | -2.66 | -3.43 | -2.12 | 4.07 |
|  | Q3 | 4.27 | 5.36 | -0.64 | 0.78 | 0.19 | 6.82 |
|  | IQR | 4.54 | 5.99 | 5.88 | 8.28 | 6.18 | 6.56 |
|  | Mean (SD) | 1.95 (3.21) | 2.27 (4.69) | -3.98 (5.69) | -3.47 (5.28) | -2.45 (7.66) | 3.68 (6.36) |
| Thoracic  (**enhanced**) | Q1 | -1.23 | -4.19 | -1.43 | -0.26 | -0.33 | -6.02 |
|  | Median | -0.02 | -2.61 | 1.71 | 2.43 | 2.00 | -3.31 |
|  | Q3 | 1.38 | 0.86 | 5.05 | 5.41 | 4.83 | -1.33 |
|  | IQR | 2.61 | 5.05 | 6.48 | 5.67 | 5.16 | 4.69 |
|  | Mean (SD) | -0.12 (2.24) | -2.07 (4.15) | 1.68 (4.13) | 2.41 (4.07) | 1.46 (6.69) | -3.27 (4.11) |
| Thoracolumbar / Lumbar  (original) | Q1 | -3.67 | -1.55 | -5.26 | -6.26 | -7.00 | -5.28 |
|  | Median | -1.14 | 1.90 | -1.92 | -3.00 | -3.17 | -2.53 |
|  | Q3 | 0.83 | 4.99 | 0.69 | 0.43 | -0.75 | 1.20 |
|  | IQR | 4.50 | 6.54 | 5.95 | 6.68 | 6.25 | 6.48 |
|  | Mean (SD) | -1.20 (3.27) | 1.49 (5.63) | -2.62(5.68) | -3.86 (6.41) | -4.25 (5.42) | -1.95 (6.60) |
| Thoracolumbar / Lumbar  (**enhanced**) | Q1 | -1.01 | -3.88 | -2.49 | -1.79 | -1.19 | -2.72 |
|  | Median | 0.27 | -1.46 | -0.19 | 1.43 | 1.92 | 0.09 |
|  | Q3 | 1.72 | 0.53 | 3.15 | 3.66 | 4.89 | 3.59 |
|  | IQR | 2.73 | 4.41 | 5.64 | 5.45 | 6.08 | 6.30 |
|  | Mean (SD) | 0.29 (2.10) | -1.27 (3.07) | 0.71 (4.92) | 1.393 (4.53) | 1.77 (4.60) | 0.18 (5.26) |

Abbreviation: **QMH:** Queen Mary Hospital; **DKCH:** Duchess of Kent Children's Hospital at Sandy Bay; **PUMCH:** Peking Union Medical College Hospital; **FAH:** First Affiliated Hospital, Zhejiang University School of Medicine; **QLH:** Qilu Hospital of Shandong University; **SDPH:** Shandong Provincial Hospital; **HSH:** Shanghai Huashan Hospital.

Next, Bland-Altman analyses were performed to evaluate the agreement between the predicted maximum Cobb angle and the GT values using both *enhanced* and *original* model. As shown in Figure S3, subfigure (a) – (f) present the Bland-Altman analysis results for *enhance* model, whereas subfigure (g) – (l) demonstrate the results for *original* model. We compared the 95% limits of agreement (LoA) intervals for *enhanced* and *original* models across both the internal and external datasets. The results indicate that, on the internal dataset (QMH&DKCH), the 95% LoA interval for the *enhanced* model was reduced by 2.24° compared to the *original* model. More substantial reductions were observed across external datasets, with decreases of 6.16° at PUMCH, 8.26° at FAH, 9.32° at QLH, 7.89° at SDPH, and 5.93° at HSH. These findings highlight a more pronounced improvement in the external datasets compared to the internal dataset, effectively narrowing the performance gap observed in the internal validation. This reflects the enhanced model’s greater consistency across different datasets, demonstrating improved robustness in handling imaging variations across multiple medical centres.


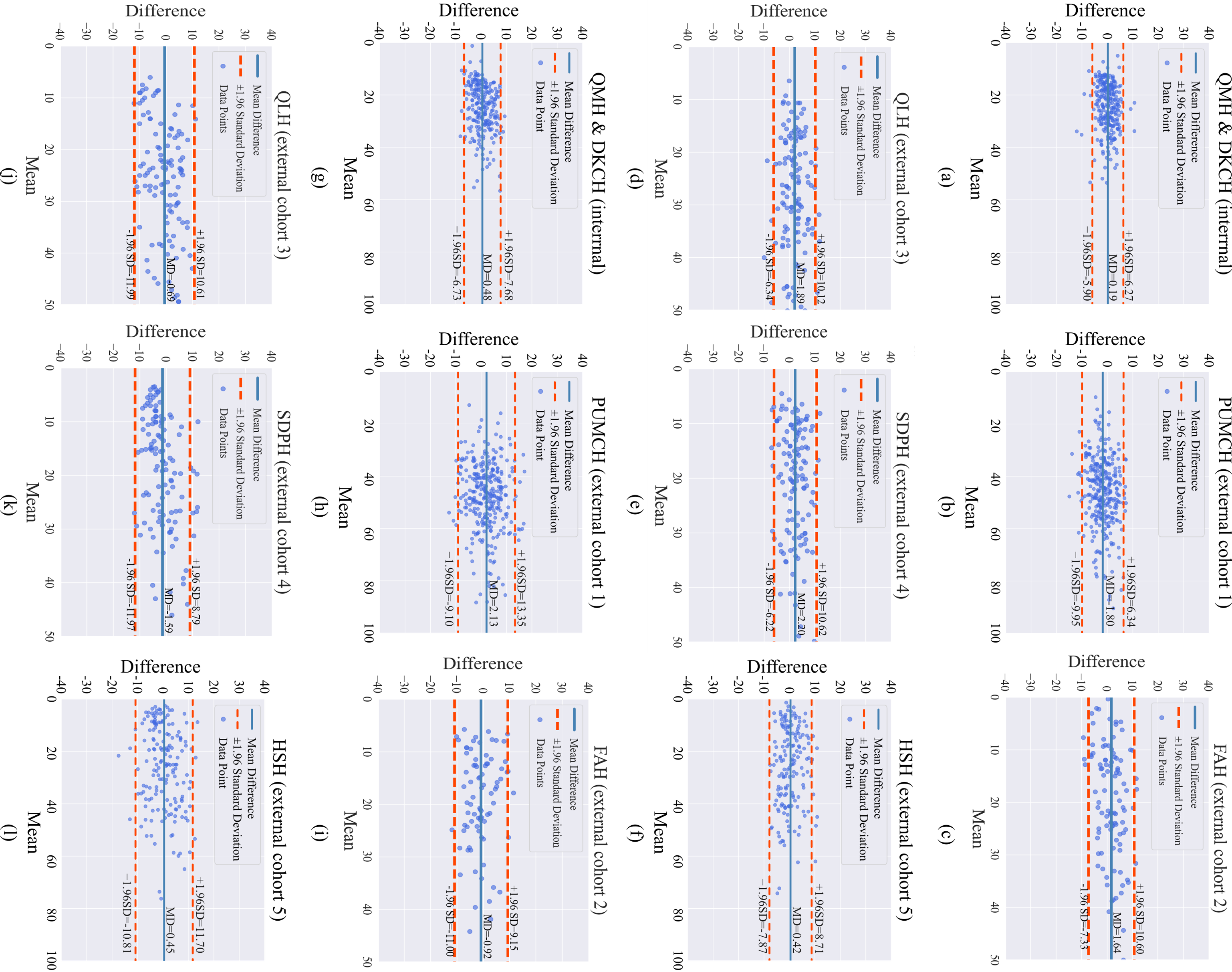


**Figure S3 |** Bland-Altman analysis for the maximum Cobb angle prediction on both internal and external validation datasets. The y-axis indicates the angle difference (in degree) between the predicted values and the GT (i.e., prediction-GT), and the x-axis represents the average degree of them (i.e., (prediction+GT)/2). Subfigure (a) – (f) illustrate the Bland-Altman analysis results for the *enhanced* model, whereas subfigure (g) – (l) present the Bland-Altman analysis results for the *original* model.

## **4.2 Performance analysis on disease severity grading.**

Figure S4 and Table S5 compare the performance of the *enhanced* and *original* model for disease severity grading. Figure S4 presents the confusion matrix analysis results for severity grading. Subfigures (a) – (f) illustrate the classification performance of the *enhanced* model across both internal and external datasets, whereas subfigures (g) – (l) depict the classification performance of the *original* model on the same datasets. More quantitative results of original model were presented in Table S5. For each dataset, we calculated five statistical measures—sensitivity, specificity, precision, negative predictive value (NPV), and accuracy—to evaluate model performance. To better assess the model’s overall ability across the three severity classes (normal-mild, moderate, and severe), we computed macro-averaged values for these statistics, defined as follows:

$$Sensitivity=Sn=\frac{\mathrm{TP}}{TP+FN} , (3)$$

$$Specificity=Sp=\frac{\mathrm{TN}}{TN+FP} , (4)$$

$$Precision=Pr=\frac{\mathrm{TP}}{TP+FP} , (5)$$

$$NPV=\frac{\mathrm{TN}}{TN+FN} , (6)$$

$$Accuracy=Acc=\frac{TP+TN}{TP+TN+FP+FN} , (7)$$

The macro-averaged value of each statistic is the mean of its values for all three severity classes, for example, the macro average of sensitivity can be calculated as:

$$\mathrm{Sensitivity}_{macro avg}=\frac{\mathrm{Sn}_{normal-mild}+\mathrm{Sn}_{moderate}+\mathrm{Sn}_{severe}}{3} (8)$$

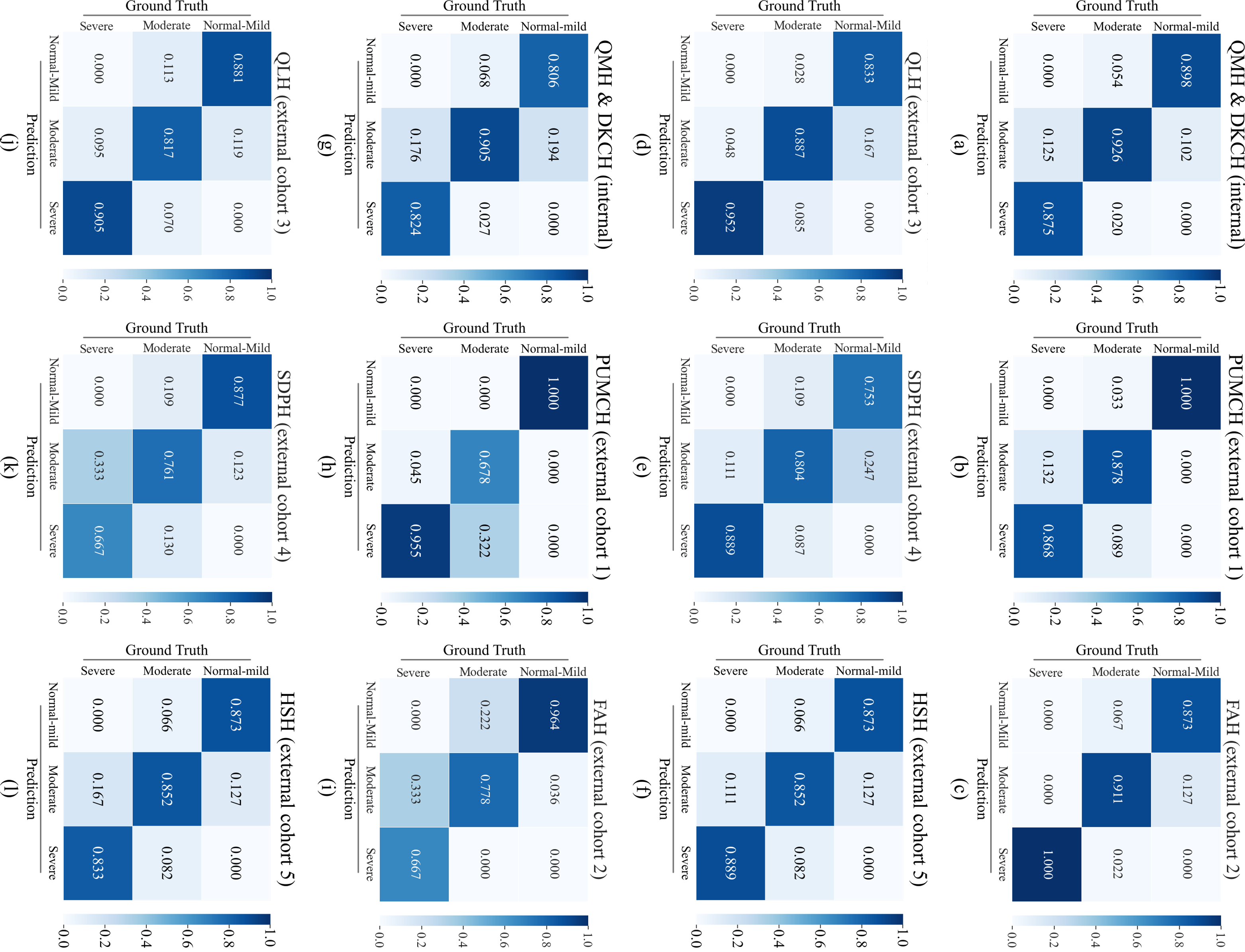


**Figure S4.** The performance comparison of *enhanced* model and *original* model on severity grading in terms of confusion matrices. All patients are classified into three groups, namely, normal-mild, moderate, and severe according to the CA value. Subfigure (a) – (f) display the confusion matrix analysis results for the *enhanced* model on internal and external datasets, whereas subfigure (g) – (l) present the confusion matrix analysis results for the *original* model on internal and external datasets.

**Table S5.**  Quantitative performance on severity grading and curve type detection using *original model*.

| **Evaluation metrics** | **Severity level** | | | | **Curve type** | | |
| --- | --- | --- | --- | --- | --- | --- | --- |
|  | **Normal-Mild**  **(0–20°)** | **Moderate**  **(21–40°)** | **Severe**  **(>41°)** | **Macro Avg.** | **T** | **TL/L** | **Macro Avg.** |
| **Internal validation dataset (QMH&DKCH cohort)** | | | | | | | |
| Sensitivity (95% CI) | 84.85%  (76.50%~90.60%) | 91.16%  (85.46%~94.76%) | 82.35%  (58.97%~93.81%) | 86.12% | 98.99%  (96.39%~99.72%) | 98.59%  (92.44%~99.75%) | 98.79% |
| Specificity (95% CI) | 94.51%  (89.90%~97.09%) | 84.48%  (76.80%~89.95%) | 98.37%  (95.89%~99.37%) | 92.46% | 98.46%  (91.79%~99.73%) | 93.75%  (89.39%~96.39%) | 96.11% |
| Precision (95% CI) | 90.32%  (82.62%~94.82%) | 88.16%  (82.06%~92.38%) | 77.78%  (54.79%~91.00%) | 85.42% | 99.49%  (97.18%~99.91%) | 85.37%  (76.14%~91.43%) | 92.43% |
| NPV  (95% CI) | 91.18%  (85.95%~94.58%) | 88.29%  (80.99%~93.03%) | 98.78%  (96.46%~99.58%) | 92.75% | 96.97%  (89.61%~99.17%) | 99.45%  (96.94%~99.90%) | 98.21% |
| Accuracy (95% CI) | 90.87%  (86.78%~93.79%) | 88.21%  (83.75%~91.57%) | 97.34%  (94.61%~98.70%) | 92.14% | 98.86%  (96.70%~99.61%) | 95.06%  (91.73%~97.09%) | 96.96% |
| **External validation dataset 1 (PUMCH cohort)** | | | | | | | |
| Sensitivity (95% CI) | 75.00%  (30.06%~95.44%) | 73.86%  (63.82%~81.91%) | 90.09%  (85.45%~93.36%) | 79.65% | 98.95%  (96.97%~99.64%) | 96.77%  (83.81%~99.43%) | 97.86% |
| Specificity (95% CI) | 100.00%  (98.78%~100.00%) | 89.82%  (85.19%~93.12%) | 75.00%  (65.27%~82.72%) | 88.27% | 100.00%  (87.54%~100.00%) | 98.94%  (96.93%~99.64%) | 99.47% |
| Precision (95% CI) | 100.00%  (43.85%~100.00%) | 73.86%  (63.82%~81.91%) | 89.69%  (85.00%~93.03%) | 87.85% | 100.00%  (98.67%~100.00%) | 90.91%  (76.43%~96.86%) | 95.45% |
| NPV  (95% CI) | 99.68%  (98.20%~99.94%) | 89.82%  (85.19%~93.12%) | 75.82%  (66.10%~83.46%) | 88.44% | 90.00%  (74.38%~96.54%) | 99.64%  (98.01%~99.94%) | 94.82% |
| Accuracy (95% CI) | 99.68%  (98.22%~99.94%) | 85.35%  (81.01%~88.83%) | 85.67%  (81.36%~89.11%) | 90.23% | 99.04%  (97.23%~99.67%) | 98.73%  (96.77%~99.50%) | 98.89% |
| **External validation dataset 2 (FAH cohort)** | | | | | | | |
| Sensitivity (95% CI) | 96.36%  (87.68%~99.00%) | 77.78%  (63.73%~87.46%) | 66.67%  (20.77%~93.85%) | 72.22% | 76.10%  (63.37%~86.86%) | 70.71%  (52.41%~86.43%) | 70.19% |
| Specificity (95% CI) | 79.17%  (65.74%~88.27%) | 94.83%  (85.86%~98.23%) | 100.00%  (96.30%~100.00%) | 97.41% | 79.55%  (65.50%~88.85%) | 70.00%  (58.69%~80.34%) | 74.77% |
| Precision (95% CI) | 84.13%  (73.19%~91.14%) | 92.11%  (79.20%~97.28%) | 100.00%  (34.24%~100.00%) | 96.05% | 81.25%  (68.06%~89.81%) | 56.17%  (43.97%~70.46%) | 68.71% |
| NPV  (95% CI) | 95.00%  (83.50%~98.62%) | 84.62%  (73.94%~91.42%) | 99.01%  (94.60%~99.83%) | 91.81% | 73.64%  (60.42%~85.07%) | 80.36%  (68.16%~88.66%) | 77.00% |
| Accuracy (95% CI) | 88.35%  (80.73%~93.21%) | 87.38%  (79.60%~92.47%) | 99.03%  (94.70%~99.83%) | 93.20% | 81.84%  (72.49%~89.62%) | 70.19%  (60.54%~79.12%) | 76.02% |
| **External validation dataset 3 (QLH cohort)** | | | | | | | |
| Sensitivity (95% CI) | 88.10%  (75.00%~94.81%) | 81.69%  (71.15%~88.98%) | 90.48%  (71.09%~97.35%) | 86.08% | 76.10%  (61.04%~90.11%) | 80.09%  (70.85%~87.95%) | 78.05% |
| Specificity (95% CI) | 91.30%  (83.77%~95.53%) | 88.89%  (78.80%~94.51%) | 95.58%  (90.06%~98.10%) | 92.23% | 73.76%  (63.68%~83.55%) | 86.67%  (67.82%~99.21%) | 80.21% |
| Precision (95% CI) | 82.22%  (68.67%~90.71%) | 89.23%  (79.40%~94.68%) | 79.17%  (59.53%~90.76%) | 84.20% | 64.85%  (54.48%~76.89%) | 89.29%  (80.88%~94.26%) | 77.07% |
| NPV  (95% CI) | 94.38%  (87.51%~97.58%) | 81.16%  (70.39%~88.65%) | 98.18%  (93.61%~99.50%) | 89.67% | 93.53%  (81.99%~100.00%) | 54.79%  (43.42%~65.69%) | 74.16% |
| Accuracy (95% CI) | 90.30%  (84.11%~94.24%) | 85.07%  (78.07%~90.13%) | 94.78%  (89.61%~97.45%) | 89.93% | 74.48%  (66.04%~82.67%) | 88.38%  (80.15%~95.60%) | 81.43% |
| **External validation dataset 4 (SDPH cohort)** | | | | | | | |
| Sensitivity (95% CI) | 87.65%  (78.74%~93.15%) | 76.09%  (62.06%~86.09%) | 66.67%  (35.42%~87.94%) | 71.38% | 65.79%  (49.89%~78.79%) | 61.63%  (51.06%~71.20%) | 63.71% |
| Specificity (95% CI) | 90.91%  (80.42%~96.05%) | 85.56%  (76.84%~91.36%) | 95.28%  (90.08%~97.82%) | 90.42% | 64.29%  (54.43%~73.07%) | 80.00%  (66.96%~88.76%) | 72.14% |
| Precision (95% CI) | 93.42%  (85.51%~97.16%) | 72.92%  (59.00%~83.43%) | 50.00%  (25.38%~74.62%) | 61.64% | 51.67%  (40.06%~64.27%) | 84.13%  (73.19%~91.14%) | 67.90% |
| NPV  (95% CI) | 83.33%  (71.97%~90.69%) | 87.50%  (78.99%~92.87%) | 97.58%  (93.13%~99.17%) | 92.54% | 82.89%  (72.90%~89.72%) | 54.79%  (43.42%~65.69%) | 68.84% |
| Accuracy (95% CI) | 88.97%  (82.60%~93.20%) | 82.35%  (75.08%~87.84%) | 93.38%  (87.90%~96.48%) | 87.87% | 64.71%  (56.37%~72.23%) | 68.38%  (60.15%~75.60%) | 66.54% |
| **External validation dataset 5 (HSH cohort)** | | | | | | | |
| Sensitivity (95% CI) | 83.54%  (73.85%~90.12%) | 72.13%  (59.83%~81.81%) | 80.56%  (64.97%~90.25%) | 78.74% | 98.84%  (93.70%~99.79%) | 89.36%  (77.41%~95.37%) | 94.10% |
| Specificity (95% CI) | 93.81%  (87.16%~97.13%) | 82.61%  (74.66%~88.45%) | 92.14%  (86.48%~95.56%) | 89.52% | 94.44%  (87.65%~97.60%) | 85.27%  (78.14%~90.36%) | 89.86% |
| Precision (95% CI) | 91.67%  (82.99%~96.12%) | 68.75%  (56.61%~78.77%) | 72.50%  (57.17%~83.89%) | 77.64% | 94.44%  (87.65%~97.60%) | 68.85%  (56.41%~79.06%) | 81.65% |
| NPV  (95% CI) | 87.50%  (79.78%~92.55%) | 84.82%  (77.03%~90.30%) | 94.85%  (89.76%~97.48%) | 89.06% | 98.84%  (93.70%~99.79%) | 95.65%  (90.22%~98.13%) | 97.24% |
| Accuracy (95% CI) | 89.20%  (83.76%~92.98%) | 78.98%  (72.37%~84.35%) | 89.77%  (84.41%~93.43%) | 85.98% | 96.59%  (92.76%~98.43%) | 86.36%  (80.51%~90.66%) | 91.48% |

*** T:** Thoracic; **TL:** Thoracolumbar; **L:** Lumbar; **NPV**: negative predictive value; **CI**: confidence interval.
